# Expression of housekeeping genes varies depending on mevalonate pathway inhibition in cancer cells

**DOI:** 10.1016/j.heliyon.2023.e18017

**Published:** 2023-07-08

**Authors:** Nanami Irie, Katsuhiko Warita, Jiro Tashiro, Yaxuan Zhou, Takuro Ishikawa, Zoltán N. Oltvai, Tomoko Warita

**Affiliations:** a Graduate School of Science and Technology, Kwansei Gakuin University, 1 Gakuen Uegahara, Sanda, Hyogo 669‐1330, Japan; b Department of Biomedical Sciences, School of Biological and Environmental Sciences, Kwansei Gakuin University, 1 Gakuen Uegahara, Sanda, Hyogo 669‐1330, Japan; c Department of Veterinary Anatomy, School of Veterinary Medicine, Tottori University, 4-101 Koyama Minami, Tottori, Tottori 680-8553, Japan; d Department of Anatomy, School of Medicine, Aichi Medical University, 1-1 Yazakokarimata, Nagakute, Aichi 480-1195, Japan; e Department of Pathology and Laboratory Medicine, University of Rochester, 601 Elmwood Ave., Rochester, NY 14642, USA

**Keywords:** Mevalonate pathway, Cancer cells, Statins, Housekeeping genes, RT-qPCR, Optimal internal reference genes

## Abstract

Statins have anticancer effects and may be used as anticancer agents via drug repositioning. In reverse transcription-quantitative polymerase chain reaction (RT-qPCR) assays, the internal reference gene must not be affected by any experimental conditions. As statins exert a wide range of effects on cells by inhibiting the mevalonate pathway, it is possible that statin treatment might alter the expression of housekeeping genes used as internal reference genes, thereby misleading the assessment of obtained gene expression data. Here, we evaluated the expression stability of internal reference genes in atorvastatin-treated cancer cell lines. We treated both statin-sensitive and statin-resistant cancer cell lines with atorvastatin at seven different concentrations and performed RT-qPCR on 15 housekeeping genes whose expression stability was then assessed using five different algorithms. In both statin-sensitive and statin-resistant cancer cell lines, atorvastatin affected the expression of certain internal reference genes in a dose-dependent and cancer cell line-dependent manner; therefore, caution should be exercised when comparing target gene expression between cells. Our findings emphasize the importance of the validation of internal reference genes in gene expression analyses in drug treatment-based cancer research.

## 1 Introduction

Quantitative analysis of gene expression in cells and tissues using reverse transcription-quantitative polymerase chain reaction (RT-qPCR) is a routine and indispensable research approach. RT-qPCR is a highly sensitive assay, but its accuracy and reliability depend on the selection of optimal reference genes for normalization. Generally, housekeeping genes whose expression levels are stable are used as reference genes to normalize mRNA expression levels among samples.

In recent years, numerous studies have shown that the mRNA expression of housekeeping genes fluctuates among samples, and information regarding potential stable reference genes that are optimal in each experimental system has been reported [[1], [2], [3], [4], [5], [6], [7], [8], [9]]. Notably, only a few studies have focused on reference gene selection in experiments wherein external agents were used. For example, five different drug or chemical treatments (paclitaxel, gossypol, methyl jasmonate, l-nicotine, and melamine) of MCF-7 breast cancer cells altered the expression patterns of genes that are considered to be expressed stably, and it was demonstrated that frequently used reference genes such as 18S ribosomal RNA, *ACTB*, and *GAPDH* are not reliable reference genes [10]. In addition, in the evaluation of the effects of hydrogen peroxide on human umbilical vein endothelial cells, 18S ribosomal RNA, a commonly used internal control, was reportedly the least stable gene among the 15 reference genes evaluated [11].

Statins are drugs used to treat dyslipidemia by specifically inhibiting 3-hydroxy-3-methyl-glutaryl coenzyme A (HMG-CoA) reductase (HMGCR), which acts as a rate-limiting factor in the mevalonate pathway of cholesterol synthesis [12]. Owing to advances in drug repositioning, statins have emerged as anticancer agents [13,14]. As the mevalonate pathway produces not only cholesterol but also intermediate metabolites involved in the lipid modification of small G proteins and the electron transport chain [12], statins exert a wide range of effects on cells and may therefore affect the expression of internal standards that require constant expression levels, leading to misreading of gene expression data [15,16]. However, to the best of our knowledge, the optimal reference genes for statin treatment experiments in cancer cells have not yet been identified.

In this study, using cancer cells treated with low to high concentrations of atorvastatin, the expression stability of 15 internal reference genes was evaluated to determine the optimum internal standard in the statin-treated experimental system using five algorithms (geNorm, BestKeeper, NormFinder, RefFinder, and the ΔCt method).

## 2 Materials and methods

### 2.1 Cell culture

In our previous research, we classified 14 types of cancer cell lines from the NCI-60 cancer cell panel sourced from the DCTD Tumor Repository (National Cancer Institute, Frederick, MD, USA) into statin-sensitive and statin-resistant cancer cells [17]. In this study, we used six of these cancer cell lines (lung cancer: HOP-92 and NCI-H322M; prostate cancer: PC-3 and DU-145; melanoma: SK-MEL-5 and MDA-MB-435). They were cultured in RPMI 1640 medium (Thermo Fisher Scientific, Waltham, MA, USA) supplemented with 10% heat-inactivated fetal bovine serum (Biosera, Boussens, France) and penicillin/streptomycin (Fujifilm Wako Pure Chemical, Osaka, Japan; final concentration: 100 units/mL penicillin G and 100 μg/mL streptomycin) in a humidified incubator at 37 °C with 5% CO_2_.

Atorvastatin (Sigma-Aldrich, St. Louis, MO, USA) dissolved in dimethyl sulfoxide (DMSO, Fujifilm Wako Pure Chemical; final concentration of 0.1% in RPMI 1640 medium) was used. The cells were seeded in six-well plates at a density of 1 × 10^5^ cells/mL, incubated overnight prior to treatment with 0.1–30 μM atorvastatin for 24 h, and then collected for RT-qPCR. Each cancer cell line treated with 0.1% DMSO served as vehicle control (0 μM). Cell cultures were conducted in triplicate. The cells were imaged using an Olympus IX71 microscope (Olympus, Tokyo, Japan).

### 2.2 RNA extraction and quantitative RT-PCR

An ISOSPIN Cell & Tissue RNA kit (Nippon Gene, Tokyo, Japan) was used to extract the total RNA from the cells 24 h after the addition of atorvastatin in accordance with the manufacturer's instructions. The RNA samples were treated with RNase-free DNase to eliminate genomic DNA contamination. The total RNA concentration was measured using a NanoDrop 2000c spectrophotometer (Thermo Fisher Scientific). The isolated RNA with an A260/280 ratio of 2.05–2.08 was used in this experiment.

cDNA was synthesized from 1 μg of RNA using the ReverTra Ace® qPCR RT Master Mix kit (Toyobo, Osaka, Japan) in triplicate. The expression levels of the 15 reference genes were examined using the Human Housekeeping Gene Primer Set (Code#3790; Takara Bio, Otsu, Japan), which has already been used to assess suitable reference genes for RT-qPCR studies [6]. Information regarding the reference genes used in this study is presented in Table 1.

**Table 1:** Information on the genes used in this analysis.

Symbol	Gene Name	Accession number	Encoded product and its function
*ATP5F1*	ATP synthase peripheral stalk-membrane subunit b	NM_001688	A subunit of mitochondrial ATP synthase, which catalyzes ATP synthesis
*TFRC*	Transferrin receptor	NM_003234	A cell surface receptor necessary for cellular iron uptake via receptor-mediated endocytosis
*YWHAZ*	Tyrosine 3-monooxygenase/tryptophan 5-monooxygenase activation protein zeta	NM_145690	A member of the 14-3-3 family of proteins, which mediate signal transduction by binding to phosphoserine-containing proteins
*RPLP0*	Ribosomal protein lateral stalk subunit P0	NM_053275	One of the acidic ribosomal proteins, which recruit translation factors and other ribosomal proteins
*RPLP1*	Ribosomal protein lateral stalk subunit P1	NM_213725	One of the acidic ribosomal proteins, which recruit translation factors and other ribosomal proteins
*RPLP2*	Ribosomal protein lateral stalk subunit P2	NM_001004	One of the acidic ribosomal proteins, which recruit translation factors and other ribosomal proteins
*ACTB*	Actin beta	NM_001101	One of the highly conserved proteins involved in cell motility, structure, integrity, and intercellular signaling
*HPRT1*	Hypoxanthine phosphoribosyltransferase 1	NM_000194	A transferase that plays a central role in the generation of purine nucleotides through the purine salvage pathway
*B2M*	Beta-2-microglobulin	NM_004048	A serum protein found in association with the major histocompatibility complex class I heavy chain on the surface of nearly all nucleated cells
*RPS18*	Ribosomal protein S18	NM_022551	A ribosomal protein that is a component of the 40S subunit
*TBP*	TATA-box binding protein	NM_003194	TATA-binding protein, which plays a pivotal intermediary role in transcriptional activation and deactivation
*PGK1*	Phosphoglycerate kinase 1	NM_000291	A glycolytic enzyme that catalyzes the conversion of 1,3-diphosphoglycerate to 3-phosphoglycerate
*PPIA*	Peptidylprolyl isomerase A	NM_021130	A member of the peptidyl-prolyl *cis*-trans isomerase (PPIase) family, which catalyzes the *cis*-trans isomerization of proline imidic peptide bonds in oligopeptides and accelerates the folding of proteins
*GAPDH*	Glyceraldehyde-3-phosphate dehydrogenase	NM_002046	A member of the glyceraldehyde-3-phosphate dehydrogenase protein family, which catalyzes an important energy-yielding step in carbohydrate metabolism
*GUSB*	Glucuronidase beta	NM_000181	A hydrolase that degrades glycosaminoglycans, including heparan sulfate, dermatan sulfate, and chondroitin-4,6-sulfate

PCR was conducted using the Applied Biosystems™ PowerUp™ SYBR™ Green Master Mix and QuantStudio® 3 Real-Time PCR System (Thermo Fisher Scientific). The thermocycling conditions were as follows: 2 min at 50 °C and 2 min at 95 °C, which was followed by 40 cycles at 95 °C for 1 s and 60 °C for 30 s. Each PCR amplification was performed in triplicate. The gene expression level was measured using a standard curve. Heatmaps for all internal reference genes in each cancer cell line treated with various concentrations of atorvastatin were generated using Heatmapper [18]. The following primer set was used for *HMGCR* expression analysis: forward 5′-GTTCTGAACTGGAACATGGGC-3′ and reverse 5′-TTCATCCTCCACAAGACAATGC-3′ [19].

### 2.3 Evaluation of the 15 reference genes using GeNorm, NormFinder, BestKeeper, RefFinder, and the comparative ΔCt method

The stability of the candidate reference genes was evaluated using geNorm (https://cellcarta.com/genomic-data-analysis/ [15]), NormFinder (https://www.moma.dk/software/normfinder [20]), BestKeeper (http://www.gene-quantification.de/bestkeeper.html [21]), and RefFinder (http://www.ciidirsinaloa.com.mx/RefFinder-master/ [22]) as software-based approaches based on their algorithms and using the comparative ΔCt method [23] as a ΔCt approach. These techniques are commonly used for selecting stable reference genes. The comparative ΔCt method proposed by Silver et al. [23] is briefly explained herein. The comparative ΔCt method is a simple method that compares the relative expression of “pairs of genes” within each sample. In our study, if the ΔCt value between genes remained constant among different statin concentration groups, it implied that both genes were stably expressed among the groups or that both genes were upregulated or downregulated simultaneously. However, if the ΔCt values between genes were altered among different concentration groups, it indicated that either one or both genes were variable. The expression difference (ΔCt) of all “gene pairs” between internal reference genes was calculated and ultimately ranked by determining the mean of the standard deviation of ΔCt. The gene with the least mean standard deviation is defined as the most stable gene, and it is thought that this is the reference gene not affected by statin treatment. Furthermore, the comprehensive ranking was performed using the geometric mean value of each ranking determined using five algorithms.

### 2.4 Statistical analysis

Statistical analysis was performed using the add-in statistical software BellCurve for Excel (version 3.23; Social Survey Research Information Co., Ltd., Tokyo, Japan). Dunnett's test was used to compare *HMGCR* mRNA expression level in each atorvastatin-treated group with that in the controls. Statistical significance was set at *P* < 0.05.

## 3 Results

### 3.1 Statin-sensitive cancer cells showed noticeable morphological changes after statin treatment

**Fig. 1:**
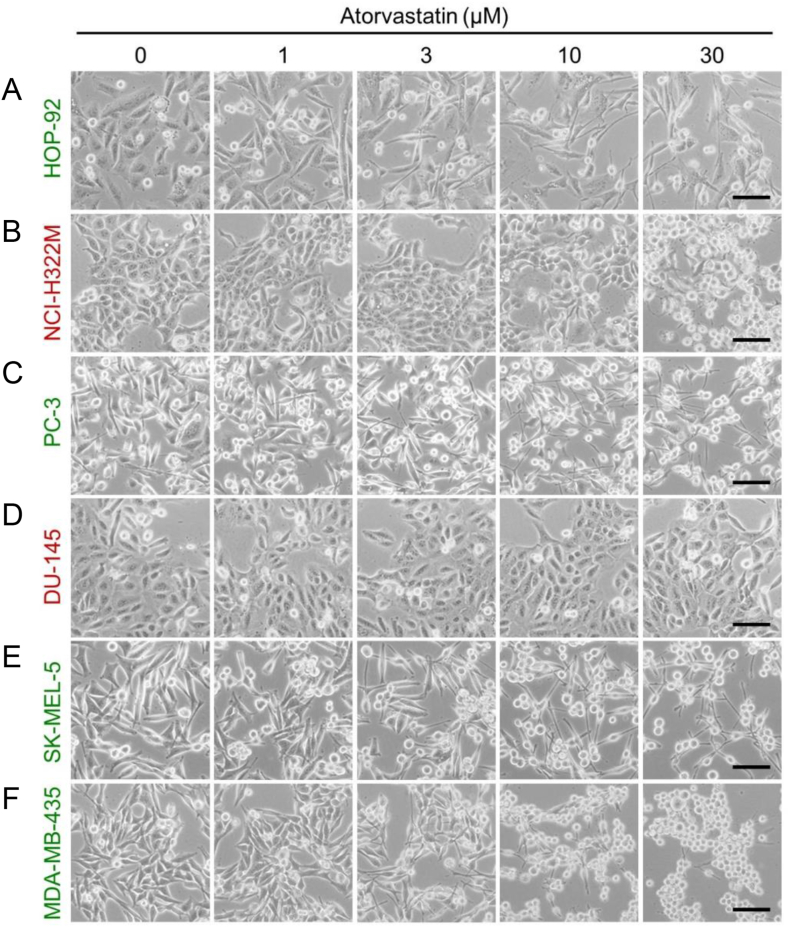
Changes in the morphology of atorvastatin-treated cells. Changes in the morphology of atorvastatin-treated HOP-92 (A), NCI-H322M (B), PC-3 (C), DU-145 (D), SK-MEL-5 (E), and MDA-MB-435 (F). Morphologies of statin-sensitive (green label) and statin-resistant (red label) cells are shown after a 24-h atorvastatin treatment period at the indicated atorvastatin concentration. Images of representative cell morphology were taken in the field of 80% confluency. Scale bar = 100 μm. (For interpretation of the references to colour in this figure legend, the reader is referred to the Web version of this article.)

Changes in cell morphology often correlate with changes in the transcriptome state of a cell [[24], [25], [26], [27]]. Therefore, we first examined the morphology of four statin-sensitive and two statin-resistant cancer cell lines before and after atorvastatin challenge. Cell morphology after 24 h of treatment with 0–30 μM atorvastatin remarkably differed between the statin-sensitive and -resistant cells. Cytoplasmic atrophy was evident in statin-sensitive cancer cells (HOP-92, PC-3, SK-MEL-5, and MDA-MB-435), and this effect was dose-dependent. By contrast, the statin-resistant NCI-H322M cells displayed only slight alterations in morphology after treatment with 30 μM atorvastatin, whereas DU-145 cells showed no remarkable changes in morphology following treatment with atorvastatin at any concentration (Fig. 1).

### 3.2 Heatmaps revealed variations in the expression of internal reference genes in statin-treated cancer cells

**Fig. 2:**
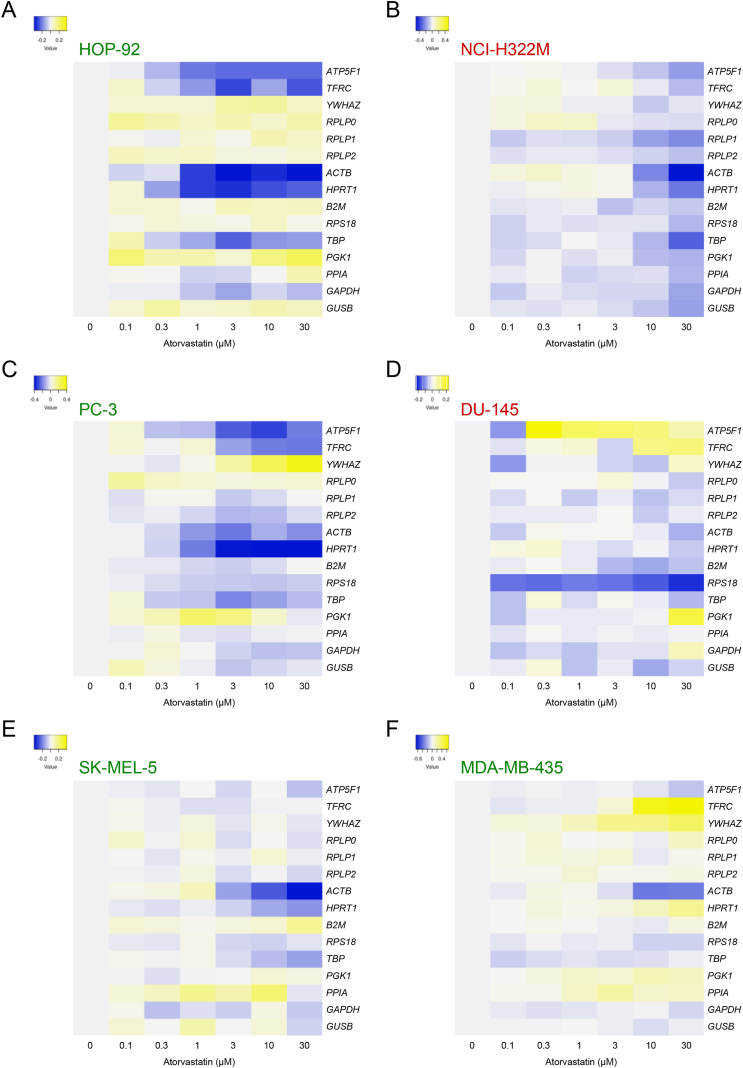
Heatmap of the gene expression levels of 15 reference genes after atorvastatin treatment. Relative gene expression levels in 0.1–30 μM atorvastatin-treated cells compared with those in vehicle-treated control (0 μM atorvastatin) lung cancer cell lines [HOP-92 (A) and NCI-H322M (B)], prostate cancer cell lines [PC-3 (C) and DU-145 (D)], and melanoma cell lines (SK-MEL-5 (E) and MDA-MB-435 (F)]. Yellow indicates a high expression of genes and blue indicates a low expression of genes compared with the expression of each reference gene of the control. Cell name in green letter and red letter indicates statin-sensitive cells and statin-resistant cells, respectively. Data are presented as the mean of triplicate samples. (For interpretation of the references to colour in this figure legend, the reader is referred to the Web version of this article.)

Next, we determined the expression of 15 frequently used reference genes in the six cancer cell lines. The threshold cycle (Ct) values are shown in Supplementary Tables S1–S6. In all cell lines, regardless of their susceptibility to statins, internal reference genes with altered expression were observed depending on the concentration of atorvastatin (Fig. 2A–F and Supplementary Fig. S1).

**Fig. 3:**
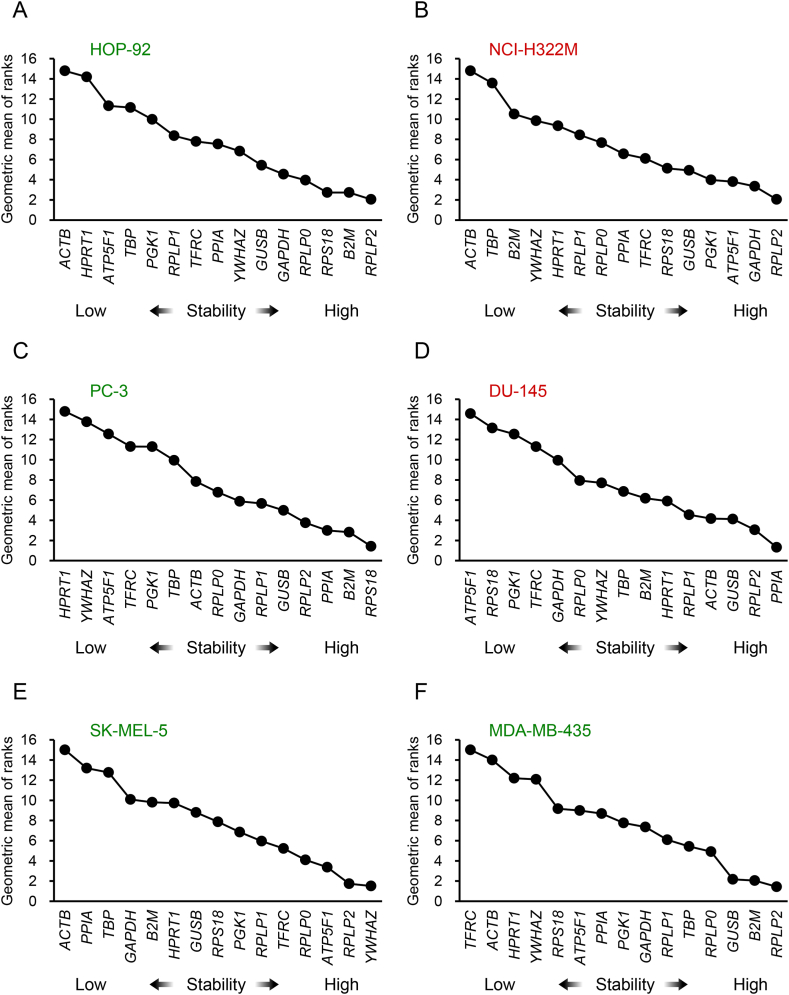
Geometric mean of rankings of reference gene stability in each cell line. Geometric mean of ranks calculated using geNorm, BestKeeper, NormFinder, RefFinder, and the ΔCt method in lung cancer cell lines [HOP-92 (A) and NCI-H322M (B)], prostate cancer cell lines [PC-3 (C) and DU-145 (D)], and melanoma cell lines [SK-MEL-5 (E) and MDA-MB-435 (F)] is shown. Cell name in green letter and red letter indicates statin-sensitive cells and statin-resistant cells, respectively. A lower value (right side of the horizontal axis) indicates a more stable reference gene. (For interpretation of the references to colour in this figure legend, the reader is referred to the Web version of this article.)

The results for lung cancer-derived statin-sensitive HOP-92 cells and statin-resistant NCI-H322M cells are shown in Fig. 3A and B, and Supplementary Table S7. Comprehensive ranking using the five algorithms yielded the same results for both cell lines, with *RPLP2* being expressed at the most stable level, whereas *ACTB* being the most unstable reference gene (Fig. 3A and B)*.* Previously, we reported that HOP-92 and NCI-H322M cells are mesenchymal- and epithelial-like cancer cells, respectively [17]. Although the cell lines have different properties, in statin-treated lung cancer cells, *RPLP2* expression was stable, whereas *ACTB* appeared to be an unstable internal reference gene.

The results for prostate cancer-derived statin-sensitive PC-3 cells are shown in Fig. 3C and Supplementary Table S8 (upper part). According to the comprehensive ranking obtained using the five algorithms, *RPS18* was the most stable gene and *HPRT1* was the least stable gene (Fig. 3C)*.*

The ranking of prostate cancer-derived statin-resistant DU-145 cells using the five algorithms is shown in Fig. 3D and Supplementary Table S8 (lower part). The comprehensive ranking using the five algorithms revealed that *PPIA* was the most stably expressed gene; however, the expression of *ATP5F1* was the least stable (Fig. 3D)*.* The expression of *ACTB*, which is less stable in statin-sensitive PC-3 cells, appeared to be less affected in DU-145 cells, which are highly resistant to atorvastatin [17,28,29].

The ranking of the internal reference genes in the melanoma-derived statin-sensitive SK-MEL-5 cells is shown in Fig. 3E and Supplementary Table S9 (upper part). *YWHAZ* was the most stable reference gene. By contrast, as in the lung cancer cell lines, *ACTB* showed the least stable expression (Fig. 3E)*.*

Fig. 3F and Supplementary Table S9 (lower part) show the ranking of the internal reference genes, as determined using the five algorithms, in the melanoma-derived statin-sensitive MDA-MB-435 cells. According to the comprehensive ranking using the five algorithms, *RPLP2* was the most stably expressed gene. By contrast, the expression of *TFRC* was the least stable (Fig. 3F)*.*

Table 2 lists the ranking of the reference genes by organ and in all cell lines. The ranking in each cancer type (lung cancer, prostate cancer, and melanoma) is based on the geometric mean of each ranking of the reference gene in two cancer cell lines. In addition, the geometric mean of each reference gene in each cancer type was further geometrically averaged and used as the basis for the comprehensive ranking among all cell lines. The comprehensive ranking results of all cancer cell lines revealed that *RPLP2* was the most stably expressed gene, followed by *B2M* and *GUSB*. By contrast, *ACTB* was the least stably expressed gene, followed by *HPRT1* and *TBP*.

**Table 2:** Ranking of reference genes used in this study according to geometric mean of ranks.

	Comprehensive ranking (All cell lines)		Lung cancer (HOP-92 & NCI-H322M)		Prostate cancer (PC-3 & DU-145)		Melanoma (SK-MEL-5 & MDA-MB-435)	
Gene	Geometric mean of ranks	Rank	Geometric mean of ranks	Rank	Geometric mean of ranks	Rank	Geometric mean of ranks	Rank
*RPLP2*	2.222	1	2.048	1	3.393	2	1.578	1
*B2M*	4.645	2	5.351	5	4.181	3	4.480	4
*GUSB*	4.678	3	5.166	4	4.536	5	4.369	3
*RPS18*	5.164	4	3.736	2	4.338	4	8.496	10
*PPIA*	5.307	5	7.025	10	1.987	1	10.707	13
*RPLP0*	5.658	6	5.500	6	7.336	8	4.488	5
*GAPDH*	6.353	7	3.893	3	7.649	9	8.613	11
*RPLP1*	6.357	8	8.399	12	5.077	6	6.025	7
*YWHAZ*	7.120	9	8.195	11	10.296	12	4.279	2
*ATP5F1*	7.875	10	6.561	8	13.530	15	5.502	6
*PGK1*	8.179	11	6.303	7	11.904	14	7.293	8
*TFRC*	8.836	12	6.887	9	11.309	13	8.859	12
*TBP*	9.456	13	12.309	14	8.255	10	8.322	9
*HPRT1*	10.544	14	11.519	13	9.345	11	10.891	14
*ACTB*	10.703	15	14.794	15	5.719	7	14.491	15

### 3.3 Gene expression of *HMGCR*, the target of statins, can be interpreted differently depending on the selection of internal reference genes

**Fig. 4:**
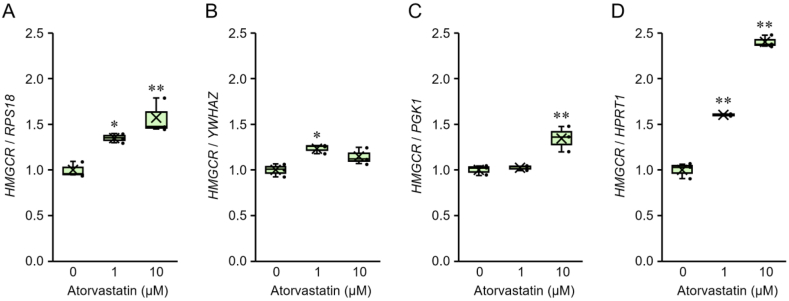
Expression of 3-hydroxy-3-methyl-glutaryl coenzyme A (HMG-CoA) reductase (*HMGCR*) normalized by the level of reference genes with different expression patterns. *HMGCR* levels in prostate cancer-derived statin-sensitive PC-3 cells treated with atorvastatin for 24 h were normalized by *RPS18* (A), *YWHAZ* (B), *PGK1* (C), and *HPRT1* (D) levels. The boxes represent the distribution of *HMGCR* levels between the first quartile (Q1) and third quartile (Q3). The horizontal line between Q1 and Q3 represents the median (Q2). “×” indicates the average value of *HMGCR* levels. The dots show individual data points. Measurement values of the experimental group were compared with the values of a control using the Dunnett's test. *P < 0.05, **P < 0.01, with respect to each control group.

Graphs of the expression of *HMGCR* specifically inhibited by statins are shown in Fig. 4. Different results were obtained when using unstable internal reference genes (Fig. 4B, C, and D), compared with the normalization results obtained with a stable internal reference gene (Fig. 4A).

## 4 Discussion

As the use of internal reference genes to normalize target genes is based on the assumption that internal reference genes are stably expressed, it is important to ensure that these internal reference genes themselves are not affected by experimental conditions [30]. However, unlike that in the conventional semi-quantitative PCR method, the subtle variation of the expression level of the internal reference gene can be detected using qPCR [30,31].

In this study, we showed that regardless of the sensitivity to statins, fluctuations in the expression of internal reference genes were evident in all cell lines depending on the concentration of atorvastatin used. This revealed the existence of certain internal reference genes whose expression was affected by statins and differed among cell types, suggesting that caution should be exercised when comparing gene expression among cancer cell lines. The commonly known ones are *ACTB* and *GAPDH*, which are used for normalizing the data via RT-qPCR without particular validation. In addition to cholesterol, the mevalonate pathway produces substances involved in the prenylation of small G proteins as intermediate products [32,33]. The Rho family of small G proteins plays a central role in regulating the intracellular actin cytoskeleton [[34], [35], [36], [37], [38]]. Therefore, statins may have an effect on actin through the inhibition of Rho prenylation. In addition, the small G protein Ras is involved in the regulation of glycolysis [39]. In other words, statins have the potential to affect *ACTB* and *GAPDH*, whose expression is thought to be subconsciously unaltered. To the best of our knowledge, there have been no reports of optimal internal reference genes in experiments to examine the anticancer effect of atorvastatin.

Fig. 4 shows a typical pattern that leads to misinterpretation of gene expression. The graph normalized to the level of *RPS18*, which is the most stable internal reference gene in PC-3 cells, indicates the actual expression level of *HMGCR* (Fig. 4A). The graph normalized to the level of *HPRT1*, which is the least stable internal reference gene in PC-3 cells, appears correct at first glance (Fig. 4D).

However, as the expression of *HPRT1* decreases in a dose-dependent manner, *HMGCR* expression is falsely inflated in a dose-dependent manner according to the statin concentration, resulting in false data (*HMGCR* expression at 10 μM atorvastatin in Fig. 4D is approximately 1.5 times higher than that in Fig. 4A). Similarly, the graph normalized to the level of *YWHAZ*, whose expression increased in a dose-dependent manner, also presents a false result (Fig. 4B). This is because the expression of *HMGCR* is canceled as the statin concentration increases. Another graph (Fig. 4C) was normalized to the level of *PGK1*, whose expression increased and decreased at low and high concentrations of statins, respectively, in a dose-dependent manner; this graph shows that the gene expression of *HMGCR* decreased and increased in a dose-dependent manner, resulting in incorrect data. Therefore, caution must be exercised when performing gene expression analysis in statin-based cancer studies. In line with our findings, Nakayama et al. [6] have shown that the expression pattern of the select target genes (IL-5, CCL11, IFN-γ, and IL-17A) differs based on the internal reference gene used. The genes exhibited similar expression patterns when normalized using the most stable experimental condition, *RPLP0* and *RPLP1*. However, when the less stable *ACTB* and *GAPDH* were used, different variations in the gene expression were observed.

The results of the present study suggest that the expression of housekeeping genes was altered by atorvastatin and that the patterns of gene expression affected by atorvastatin were different for various cells. Internal reference genes must be carefully selected to avoid misleading results due to the misinterpretation of data; therefore, the optimal reference gene used in one cell line may be inappropriate for another cell line, and the best reference gene should be selected on a case-by-case basis. The stability ranking of internal reference genes obtained in this study provides guidance for gene expression analysis in cancer research using statins and offers valuable information. However, as RT-qPCR data only evaluate the ratio of compositions in a given amount of total RNA (1 μg in this study), it is difficult to determine whether a gene whose expression remains ''unchanged'' is truly constant or is ''seemingly unchanged'' because the expression of other genes is also changing. This is a very prominent limitation. In addition, the approach used in this study for stability evaluation was not exhaustive; other equivalence tests have also been proposed [40,41], and if used in combination, more reliable data may be produced.

In conclusion, our findings emphasize the importance of validating internal reference genes in gene expression analyses in drug treatment-based cancer research. Furthermore, while we employed five commonly used analyses to evaluate the optimal internal reference genes, it is important to note that they do not represent all available methods [40,41].

## Authorship contribution statement

Katsuhiko Warita, Tomoko Warita: Concieved and designed the experiments; Performed the experiments; Analyzed and interpreted the data; Contributed reagents, materials, analysis tools or data; Wrote the paper.

Nanami Irie: Performed the experiments; Analyzed and interpreted the data; Wrote the paper.

Jiro Tashiro, Yaxuan Zhou, Takuro Ishikawa: Performed the experiments.

Zoltán N. Oltvavi: Wrote the paper.

## Data availability statement

All relevant data supporting the key findings of this study are available within the article and its Supplementary Information files or from the corresponding author on reasonable request.

## Declaration of competing interest

The authors declare that they have no known competing financial interests or personal relationships that could have appeared to influence the work reported in this paper.

## Abbreviations

RT-qPCR: reverse transcription-quantitative polymerase chain reaction
HMGCR: 3-hydroxy-3-methyl-glutaryl-coenzyme A reductase
