# Supplementary material for: Expression of housekeeping genes varies depending on mevalonate pathway inhibition in cancer cells
Source: Heliyon. 2023 Jul 8;9(7):e18017. doi: 10.1016/j.heliyon.2023.e18017 (PMC10368838; doi:10.1016/j.heliyon.2023.e18017)
Supplement: Multimedia component 1 [file mmc1.docx]

Table S1. The Ct values of lung cancer-derived statin-sensitive HOP-92 cell line

|  |  | Reference gene | | | | | | | | | | | | | | |
| --- | --- | --- | --- | --- | --- | --- | --- | --- | --- | --- | --- | --- | --- | --- | --- | --- |
|  |  | *ATP5F1* | *TFRC* | *YWHAZ* | *RPLP0* | *RPLP1* | *RPLP2* | *ACTB* | *HPRT1* | *B2M* | *RPS18* | *TBP* | *PGK1* | *PPIA* | *GAPDH* | *GUSB* |
| Concentration of atorvastatin | 0 µM | 20.14 | 20.99 | 18.89 | 17.47 | 15.74 | 18.05 | 14.77 | 22.36 | 19.07 | 17.43 | 24.90 | 20.86 | 16.43 | 16.43 | 23.04 |
|  |  | 20.16 | 21.00 | 18.70 | 17.38 | 15.84 | 18.04 | 14.62 | 22.30 | 19.01 | 17.40 | 24.71 | 20.61 | 16.30 | 16.58 | 22.76 |
|  |  | 20.03 | 20.83 | 18.75 | 17.22 | 15.73 | 18.01 | 14.84 | 22.16 | 18.85 | 17.47 | 24.79 | 20.78 | 16.15 | 16.46 | 22.99 |
|  | 0.1 µM | 20.09 | 21.03 | 18.76 | 17.08 | 15.93 | 17.86 | 14.89 | 22.26 | 18.89 | 17.58 | 24.79 | 20.43 | 16.38 | 16.60 | 22.96 |
|  |  | 20.13 | 20.76 | 18.62 | 17.17 | 15.66 | 17.84 | 14.79 | 22.12 | 18.94 | 17.29 | 24.52 | 20.38 | 16.18 | 16.38 | 22.79 |
|  |  | 20.17 | 20.70 | 18.68 | 17.16 | 15.67 | 17.97 | 14.84 | 22.20 | 18.83 | 17.40 | 24.65 | 20.55 | 16.26 | 16.53 | 22.80 |
|  | 0.3 µM | 20.20 | 20.87 | 18.67 | 17.22 | 15.65 | 17.94 | 14.72 | 22.57 | 18.81 | 17.35 | 24.89 | 20.43 | 16.10 | 16.38 | 22.67 |
|  |  | 20.31 | 21.26 | 18.76 | 17.11 | 15.90 | 17.89 | 14.83 | 22.47 | 18.98 | 17.42 | 24.92 | 20.69 | 16.38 | 16.63 | 22.77 |
|  |  | 20.37 | 21.00 | 18.64 | 17.24 | 15.85 | 17.94 | 14.85 | 22.55 | 18.91 | 17.28 | 24.87 | 20.64 | 16.37 | 16.50 | 22.74 |
|  | 1 µM | 20.44 | 21.25 | 18.66 | 17.13 | 15.54 | 17.82 | 15.32 | 22.83 | 18.88 | 17.39 | 24.92 | 20.43 | 16.28 | 16.52 | 22.82 |
|  |  | 20.54 | 21.33 | 18.75 | 17.27 | 15.71 | 18.08 | 15.31 | 22.87 | 19.07 | 17.42 | 25.09 | 20.70 | 16.44 | 16.63 | 22.89 |
|  |  | 20.56 | 21.13 | 18.69 | 17.32 | 15.78 | 17.82 | 15.40 | 22.96 | 18.93 | 17.30 | 24.98 | 20.60 | 16.43 | 16.69 | 22.82 |
|  | 3 µM | 20.53 | 21.50 | 18.64 | 17.31 | 15.77 | 18.06 | 15.52 | 22.92 | 18.85 | 17.31 | 25.24 | 20.69 | 16.36 | 16.75 | 22.79 |
|  |  | 20.55 | 21.45 | 18.48 | 17.07 | 15.67 | 17.77 | 15.39 | 22.91 | 18.92 | 17.34 | 25.26 | 20.57 | 16.28 | 16.62 | 22.70 |
|  |  | 20.63 | 21.66 | 18.63 | 17.21 | 15.78 | 18.10 | 15.66 | 23.00 | 18.73 | 17.57 | 25.21 | 20.73 | 16.46 | 16.73 | 22.98 |
|  | 10 µM | 20.57 | 21.28 | 18.61 | 17.26 | 15.61 | 18.00 | 15.46 | 22.84 | 18.90 | 17.45 | 25.06 | 20.58 | 16.40 | 16.65 | 22.95 |
|  |  | 20.48 | 21.14 | 18.58 | 17.13 | 15.52 | 17.99 | 15.36 | 22.80 | 18.82 | 17.17 | 25.00 | 20.42 | 16.12 | 16.46 | 22.65 |
|  |  | 20.63 | 21.13 | 18.52 | 17.36 | 15.64 | 17.97 | 15.40 | 22.78 | 18.87 | 17.32 | 25.10 | 20.45 | 16.34 | 16.59 | 22.71 |
|  | 30 µM | 20.51 | 21.37 | 18.49 | 17.12 | 15.72 | 17.88 | 15.43 | 22.65 | 18.82 | 17.35 | 25.02 | 20.38 | 16.18 | 16.69 | 22.73 |
|  |  | 20.69 | 21.69 | 18.78 | 17.19 | 15.50 | 17.98 | 15.53 | 22.85 | 18.97 | 17.50 | 25.23 | 20.36 | 16.16 | 16.69 | 22.89 |
|  |  | 20.52 | 21.39 | 18.72 | 17.25 | 15.72 | 17.96 | 15.57 | 22.75 | 18.76 | 17.34 | 24.89 | 20.42 | 16.06 | 16.54 | 22.76 |

Table S2. The Ct values of lung cancer-derived statin-resistant NCI-H322M cell line

|  |  | Reference gene | | | | | | | | | | | | | | |
| --- | --- | --- | --- | --- | --- | --- | --- | --- | --- | --- | --- | --- | --- | --- | --- | --- |
|  |  | *ATP5F1* | *TFRC* | *YWHAZ* | *RPLP0* | *RPLP1* | *RPLP2* | *ACTB* | *HPRT1* | *B2M* | *RPS18* | *TBP* | *PGK1* | *PPIA* | *GAPDH* | *GUSB* |
| Concentration of atorvastatin | 0 µM | 20.37 | 19.63 | 18.57 | 17.80 | 15.27 | 17.84 | 14.29 | 22.58 | 21.14 | 17.63 | 23.91 | 21.84 | 16.99 | 14.87 | 22.23 |
|  |  | 20.44 | 19.62 | 18.55 | 17.82 | 15.31 | 17.75 | 14.32 | 22.63 | 21.17 | 17.61 | 23.75 | 21.79 | 17.00 | 14.98 | 22.15 |
|  |  | 20.50 | 19.83 | 18.69 | 17.70 | 15.57 | 17.73 | 14.55 | 22.75 | 21.14 | 17.74 | 24.05 | 21.98 | 17.09 | 15.07 | 22.24 |
|  | 0.1 µM | 20.50 | 19.70 | 18.44 | 17.72 | 15.48 | 17.81 | 14.34 | 22.81 | 21.42 | 17.65 | 24.24 | 21.99 | 17.17 | 15.13 | 22.29 |
|  |  | 20.48 | 19.73 | 18.57 | 17.75 | 15.56 | 18.00 | 14.32 | 22.61 | 21.09 | 17.83 | 23.88 | 21.98 | 17.00 | 15.21 | 22.31 |
|  |  | 20.32 | 19.57 | 18.61 | 17.69 | 15.66 | 17.78 | 14.29 | 22.60 | 21.15 | 17.95 | 23.95 | 21.98 | 17.05 | 15.02 | 22.21 |
|  | 0.3 µM | 20.35 | 19.48 | 18.47 | 17.52 | 15.45 | 17.78 | 14.12 | 22.58 | 21.13 | 17.73 | 23.87 | 21.86 | 17.07 | 15.04 | 22.30 |
|  |  | 20.43 | 19.62 | 18.69 | 17.55 | 15.47 | 17.83 | 14.29 | 22.65 | 21.15 | 17.72 | 24.04 | 21.88 | 16.90 | 15.01 | 22.24 |
|  |  | 20.45 | 19.69 | 18.45 | 17.80 | 15.47 | 17.85 | 14.34 | 22.64 | 21.30 | 17.69 | 24.05 | 21.87 | 17.13 | 15.00 | 22.32 |
|  | 1 µM | 20.39 | 19.58 | 18.45 | 17.64 | 15.49 | 17.70 | 14.16 | 22.56 | 21.07 | 17.64 | 23.84 | 21.83 | 16.98 | 15.03 | 22.29 |
|  |  | 20.51 | 19.67 | 18.67 | 17.61 | 15.42 | 17.88 | 14.35 | 22.60 | 21.30 | 17.82 | 23.93 | 22.05 | 17.25 | 15.04 | 22.09 |
|  |  | 20.46 | 19.81 | 18.75 | 17.67 | 15.50 | 17.86 | 14.45 | 22.61 | 21.18 | 17.77 | 23.91 | 21.96 | 17.32 | 15.10 | 22.39 |
|  | 3 µM | 20.46 | 19.56 | 18.54 | 17.78 | 15.47 | 17.78 | 14.36 | 22.59 | 21.25 | 17.67 | 23.98 | 21.87 | 17.04 | 15.07 | 22.21 |
|  |  | 20.50 | 19.60 | 18.62 | 17.87 | 15.56 | 17.84 | 14.35 | 22.61 | 21.40 | 17.76 | 23.86 | 21.84 | 17.12 | 15.04 | 22.27 |
|  |  | 20.57 | 19.65 | 18.71 | 17.78 | 15.59 | 17.95 | 14.36 | 22.69 | 21.42 | 17.74 | 24.02 | 21.97 | 17.23 | 15.12 | 22.42 |
|  | 10 µM | 20.60 | 19.69 | 18.73 | 17.87 | 15.81 | 18.00 | 14.63 | 22.98 | 21.26 | 17.67 | 24.03 | 22.14 | 17.11 | 15.03 | 22.30 |
|  |  | 20.70 | 19.74 | 18.84 | 17.91 | 15.71 | 17.92 | 14.88 | 22.96 | 21.28 | 17.91 | 24.38 | 22.19 | 17.16 | 15.17 | 22.44 |
|  |  | 20.59 | 19.77 | 18.75 | 17.77 | 15.67 | 17.84 | 15.03 | 22.79 | 21.23 | 17.63 | 24.00 | 21.93 | 17.11 | 15.05 | 22.42 |
|  | 30 µM | 20.67 | 19.87 | 18.55 | 17.74 | 16.04 | 17.98 | 15.47 | 23.25 | 21.12 | 17.96 | 24.37 | 22.01 | 17.32 | 15.20 | 22.45 |
|  |  | 20.79 | 20.03 | 18.68 | 17.83 | 15.65 | 17.99 | 15.50 | 23.13 | 21.43 | 17.88 | 24.55 | 22.13 | 17.25 | 15.16 | 22.78 |
|  |  | 20.97 | 19.89 | 18.76 | 18.07 | 15.90 | 18.04 | 15.64 | 23.25 | 21.41 | 17.92 | 24.63 | 22.25 | 17.33 | 15.47 | 22.45 |

Table S3. The Ct values of prostate cancer-derived statin-sensitive PC-3 cell line

|  |  | Reference gene | | | | | | | | | | | | | | |
| --- | --- | --- | --- | --- | --- | --- | --- | --- | --- | --- | --- | --- | --- | --- | --- | --- |
|  |  | *ATP5F1* | *TFRC* | *YWHAZ* | *RPLP0* | *RPLP1* | *RPLP2* | *ACTB* | *HPRT1* | *B2M* | *RPS18* | *TBP* | *PGK1* | *PPIA* | *GAPDH* | *GUSB* |
| Concentration of atorvastatin | 0 µM | 21.73 | 22.36 | 20.29 | 18.51 | 17.81 | 19.20 | 16.75 | 23.76 | 20.75 | 19.22 | 25.98 | 21.54 | 18.90 | 18.34 | 24.78 |
|  |  | 21.51 | 22.14 | 20.15 | 18.45 | 17.80 | 19.18 | 16.53 | 23.54 | 20.76 | 19.20 | 26.00 | 21.40 | 18.83 | 18.32 | 24.86 |
|  |  | 21.41 | 22.22 | 20.21 | 18.80 | 17.68 | 18.97 | 16.53 | 23.63 | 20.63 | 19.26 | 25.96 | 21.39 | 18.96 | 18.19 | 24.95 |
|  | 0.1 µM | 21.35 | 22.20 | 20.16 | 18.33 | 17.77 | 19.07 | 16.62 | 23.58 | 20.74 | 19.30 | 25.80 | 21.38 | 18.93 | 18.29 | 24.78 |
|  |  | 21.45 | 22.03 | 20.33 | 18.28 | 17.73 | 19.19 | 16.56 | 23.62 | 20.73 | 19.17 | 25.93 | 21.26 | 18.89 | 18.31 | 24.71 |
|  |  | 21.63 | 22.21 | 20.20 | 18.42 | 18.01 | 19.25 | 16.64 | 23.74 | 20.75 | 19.25 | 26.00 | 21.45 | 18.95 | 18.27 | 24.69 |
|  | 0.3 µM | 21.62 | 22.13 | 20.23 | 18.51 | 17.72 | 19.18 | 16.73 | 23.71 | 20.85 | 19.34 | 26.20 | 21.27 | 18.82 | 18.12 | 24.84 |
|  |  | 21.74 | 22.46 | 20.34 | 18.50 | 17.81 | 19.23 | 16.74 | 23.83 | 20.77 | 19.37 | 26.09 | 21.35 | 18.92 | 18.19 | 24.93 |
|  |  | 21.77 | 22.18 | 20.23 | 18.34 | 17.71 | 19.00 | 16.60 | 23.69 | 20.64 | 19.19 | 26.08 | 21.23 | 18.69 | 18.23 | 24.67 |
|  | 1 µM | 21.63 | 22.21 | 20.16 | 18.37 | 17.52 | 19.27 | 16.90 | 23.98 | 20.72 | 19.28 | 26.14 | 21.06 | 18.97 | 18.25 | 24.82 |
|  |  | 21.73 | 22.12 | 20.15 | 18.46 | 17.77 | 19.12 | 16.86 | 24.08 | 20.92 | 19.31 | 26.15 | 21.09 | 18.94 | 18.23 | 24.91 |
|  |  | 21.77 | 22.14 | 20.26 | 18.58 | 17.97 | 19.27 | 16.94 | 24.09 | 20.78 | 19.42 | 26.12 | 21.15 | 19.04 | 18.34 | 24.91 |
|  | 3 µM | 22.05 | 22.32 | 19.98 | 18.53 | 17.92 | 19.27 | 16.88 | 24.45 | 20.80 | 19.33 | 26.56 | 21.08 | 18.97 | 18.39 | 24.93 |
|  |  | 21.99 | 22.66 | 20.03 | 18.51 | 17.84 | 19.44 | 17.09 | 24.50 | 20.83 | 19.43 | 26.28 | 21.17 | 19.05 | 18.31 | 24.99 |
|  |  | 22.16 | 22.58 | 20.05 | 18.55 | 17.99 | 19.28 | 17.08 | 24.46 | 20.84 | 19.31 | 26.28 | 21.20 | 19.00 | 18.47 | 25.06 |
|  | 10 µM | 22.22 | 22.76 | 19.86 | 18.48 | 17.87 | 19.31 | 16.79 | 24.42 | 20.75 | 19.45 | 26.25 | 21.36 | 18.99 | 18.42 | 24.97 |
|  |  | 22.21 | 22.56 | 19.85 | 18.53 | 17.86 | 19.23 | 16.77 | 24.50 | 20.80 | 19.30 | 26.30 | 21.38 | 18.87 | 18.45 | 24.85 |
|  |  | 22.08 | 22.64 | 19.86 | 18.48 | 17.83 | 19.39 | 16.93 | 24.60 | 20.80 | 19.35 | 26.23 | 21.26 | 18.97 | 18.49 | 25.02 |
|  | 30 µM | 21.97 | 22.78 | 19.72 | 18.48 | 17.84 | 19.28 | 17.01 | 24.53 | 20.70 | 19.33 | 26.11 | 21.49 | 18.85 | 18.49 | 24.79 |
|  |  | 21.91 | 22.61 | 19.72 | 18.56 | 17.83 | 19.20 | 16.86 | 24.52 | 20.70 | 19.30 | 26.31 | 21.53 | 18.91 | 18.52 | 24.95 |
|  |  | 21.93 | 22.63 | 19.69 | 18.40 | 17.62 | 19.12 | 16.91 | 24.41 | 20.71 | 19.40 | 26.10 | 21.41 | 18.98 | 18.29 | 24.98 |

Table S4. The Ct values of prostate cancer-derived statin-resistant DU-145 cell line

|  |  | Reference gene | | | | | | | | | | | | | | |
| --- | --- | --- | --- | --- | --- | --- | --- | --- | --- | --- | --- | --- | --- | --- | --- | --- |
|  |  | *ATP5F1* | *TFRC* | *YWHAZ* | *RPLP0* | *RPLP1* | *RPLP2* | *ACTB* | *HPRT1* | *B2M* | *RPS18* | *TBP* | *PGK1* | *PPIA* | *GAPDH* | *GUSB* |
| Concentration of atorvastatin | 0 µM | 22.36 | 22.62 | 21.18 | 18.39 | 16.87 | 19.50 | 16.89 | 24.04 | 20.82 | 17.95 | 25.61 | 22.58 | 18.48 | 17.78 | 24.63 |
|  |  | 22.25 | 22.97 | 21.19 | 18.60 | 16.82 | 19.61 | 16.93 | 24.06 | 20.94 | 18.17 | 25.69 | 22.70 | 18.64 | 17.90 | 24.73 |
|  |  | 22.03 | 22.72 | 21.13 | 18.40 | 16.74 | 19.41 | 16.92 | 23.99 | 21.01 | 18.01 | 25.87 | 22.65 | 18.54 | 17.85 | 24.62 |
|  | 0.1 µM | 22.61 | 22.82 | 21.33 | 18.59 | 17.02 | 19.54 | 17.05 | 24.10 | 20.98 | 18.15 | 25.89 | 22.76 | 18.59 | 18.09 | 24.62 |
|  |  | 22.22 | 22.79 | 21.35 | 18.28 | 16.66 | 19.47 | 16.99 | 23.86 | 20.93 | 18.26 | 25.75 | 22.76 | 18.67 | 17.85 | 24.70 |
|  |  | 22.31 | 22.76 | 21.34 | 18.49 | 16.92 | 19.57 | 16.95 | 24.03 | 20.90 | 18.39 | 25.84 | 22.71 | 18.61 | 17.87 | 24.68 |
|  | 0.3 µM | 21.82 | 22.60 | 21.19 | 18.35 | 16.88 | 19.41 | 16.88 | 23.88 | 20.91 | 18.24 | 25.67 | 22.65 | 18.40 | 17.85 | 24.58 |
|  |  | 21.85 | 22.76 | 21.19 | 18.60 | 16.73 | 19.58 | 16.89 | 24.01 | 20.83 | 18.17 | 25.72 | 22.68 | 18.54 | 17.89 | 24.60 |
|  |  | 21.94 | 22.83 | 21.14 | 18.45 | 16.77 | 19.61 | 16.90 | 24.00 | 20.99 | 18.45 | 25.64 | 22.65 | 18.66 | 17.94 | 24.68 |
|  | 1 µM | 22.08 | 22.69 | 21.11 | 18.45 | 17.01 | 19.49 | 16.83 | 24.02 | 21.01 | 18.26 | 25.83 | 22.69 | 18.65 | 17.99 | 24.71 |
|  |  | 21.97 | 22.73 | 21.29 | 18.51 | 16.88 | 19.61 | 16.97 | 24.06 | 20.95 | 18.18 | 25.78 | 22.64 | 18.53 | 18.04 | 24.84 |
|  |  | 21.94 | 22.68 | 21.14 | 18.43 | 16.82 | 19.52 | 16.91 | 24.09 | 20.89 | 18.30 | 25.72 | 22.66 | 18.58 | 17.78 | 24.69 |
|  | 3 µM | 21.91 | 22.79 | 21.24 | 18.39 | 16.85 | 19.47 | 16.91 | 24.04 | 20.92 | 18.25 | 25.68 | 22.63 | 18.50 | 17.77 | 24.60 |
|  |  | 22.01 | 22.82 | 21.27 | 18.41 | 16.82 | 19.48 | 16.93 | 24.16 | 20.94 | 18.26 | 25.68 | 22.67 | 18.58 | 17.95 | 24.67 |
|  |  | 22.10 | 22.88 | 21.17 | 18.44 | 16.83 | 19.56 | 16.90 | 24.05 | 21.23 | 18.27 | 25.74 | 22.71 | 18.59 | 17.93 | 24.75 |
|  | 10 µM | 22.03 | 22.53 | 21.28 | 18.57 | 16.95 | 19.61 | 16.90 | 24.07 | 21.11 | 18.32 | 25.76 | 22.57 | 18.56 | 17.84 | 24.76 |
|  |  | 21.98 | 22.57 | 21.23 | 18.38 | 16.94 | 19.60 | 17.00 | 23.93 | 21.10 | 18.27 | 25.76 | 22.74 | 18.63 | 17.89 | 24.78 |
|  |  | 22.09 | 22.67 | 21.27 | 18.46 | 16.88 | 19.59 | 16.92 | 24.12 | 20.98 | 18.34 | 25.73 | 22.63 | 18.62 | 17.92 | 24.80 |
|  | 30 µM | 22.08 | 22.57 | 21.04 | 18.44 | 16.79 | 19.44 | 17.20 | 24.16 | 20.96 | 18.51 | 25.93 | 22.32 | 18.54 | 17.64 | 24.68 |
|  |  | 22.15 | 22.59 | 21.13 | 18.52 | 16.76 | 19.57 | 16.90 | 24.16 | 21.05 | 18.39 | 25.85 | 22.36 | 18.63 | 17.79 | 24.88 |
|  |  | 22.08 | 22.53 | 21.13 | 18.69 | 17.06 | 19.57 | 17.05 | 24.00 | 21.04 | 18.29 | 25.73 | 22.48 | 18.55 | 17.84 | 24.59 |

Table S5. The Ct values of melanoma-derived statin-sensitive SK-MEL-5 cell line

|  |  | Reference gene | | | | | | | | | | | | | | |
| --- | --- | --- | --- | --- | --- | --- | --- | --- | --- | --- | --- | --- | --- | --- | --- | --- |
|  |  | *ATP5F1* | *TFRC* | *YWHAZ* | *RPLP0* | *RPLP1* | *RPLP2* | *ACTB* | *HPRT1* | *B2M* | *RPS18* | *TBP* | *PGK1* | *PPIA* | *GAPDH* | *GUSB* |
| Concentration of atorvastatin | 0 µM | 20.97 | 20.55 | 18.82 | 18.13 | 15.80 | 18.16 | 15.30 | 23.58 | 20.98 | 17.68 | 24.92 | 21.00 | 16.80 | 16.42 | 22.99 |
|  |  | 20.98 | 20.56 | 18.88 | 18.08 | 15.84 | 18.16 | 15.30 | 23.51 | 20.80 | 17.74 | 24.80 | 21.00 | 16.72 | 16.47 | 23.05 |
|  |  | 20.91 | 20.64 | 18.76 | 17.98 | 15.82 | 18.03 | 15.16 | 23.47 | 20.74 | 17.66 | 25.05 | 20.97 | 16.79 | 16.39 | 22.99 |
|  | 0.1 µM | 21.03 | 20.67 | 18.75 | 17.91 | 15.82 | 18.09 | 15.25 | 23.55 | 20.71 | 17.76 | 24.86 | 20.98 | 16.65 | 16.45 | 22.84 |
|  |  | 20.91 | 20.51 | 18.82 | 18.01 | 15.88 | 18.15 | 15.20 | 23.62 | 20.79 | 17.62 | 24.96 | 20.95 | 16.73 | 16.39 | 22.92 |
|  |  | 20.99 | 20.52 | 18.79 | 17.94 | 15.74 | 18.10 | 15.21 | 23.51 | 20.75 | 17.79 | 24.86 | 21.07 | 16.74 | 16.36 | 22.98 |
|  | 0.3 µM | 21.09 | 20.59 | 18.81 | 18.06 | 15.84 | 18.06 | 15.17 | 23.59 | 20.80 | 17.62 | 24.88 | 21.03 | 16.75 | 16.56 | 23.01 |
|  |  | 20.93 | 20.66 | 18.89 | 18.09 | 15.93 | 18.18 | 15.19 | 23.59 | 20.79 | 17.94 | 24.94 | 21.03 | 16.71 | 16.57 | 22.99 |
|  |  | 20.99 | 20.56 | 18.81 | 18.04 | 15.82 | 18.15 | 15.28 | 23.55 | 20.75 | 17.69 | 24.94 | 21.09 | 16.53 | 16.60 | 23.01 |
|  | 1 µM | 20.85 | 20.70 | 18.72 | 18.01 | 15.81 | 18.04 | 15.11 | 23.51 | 20.75 | 17.57 | 24.87 | 21.01 | 16.53 | 16.45 | 22.89 |
|  |  | 21.04 | 20.60 | 18.80 | 17.97 | 15.86 | 18.01 | 15.14 | 23.60 | 20.86 | 17.71 | 24.84 | 20.93 | 16.57 | 16.45 | 22.91 |
|  |  | 20.95 | 20.64 | 18.77 | 17.95 | 15.69 | 18.10 | 15.14 | 23.50 | 20.83 | 17.69 | 24.94 | 21.03 | 16.56 | 16.57 | 22.72 |
|  | 3 µM | 21.05 | 20.66 | 18.85 | 18.09 | 15.85 | 18.11 | 15.50 | 23.59 | 20.78 | 17.75 | 24.97 | 20.86 | 16.69 | 16.52 | 23.02 |
|  |  | 21.01 | 20.58 | 18.79 | 18.11 | 15.77 | 18.23 | 15.45 | 23.69 | 20.72 | 17.72 | 24.90 | 20.97 | 16.55 | 16.50 | 22.91 |
|  |  | 21.07 | 20.70 | 18.94 | 18.19 | 15.90 | 18.23 | 15.50 | 23.60 | 20.82 | 17.89 | 25.15 | 21.13 | 16.65 | 16.62 | 23.06 |
|  | 10 µM | 20.98 | 20.63 | 18.88 | 18.07 | 15.91 | 18.18 | 15.76 | 23.73 | 20.77 | 17.77 | 25.18 | 20.99 | 16.56 | 16.50 | 22.88 |
|  |  | 20.88 | 20.51 | 18.76 | 17.96 | 15.56 | 18.04 | 15.63 | 23.79 | 20.69 | 17.82 | 24.98 | 20.84 | 16.44 | 16.32 | 22.95 |
|  |  | 20.97 | 20.64 | 18.72 | 18.16 | 15.71 | 18.13 | 15.77 | 23.75 | 20.73 | 17.83 | 25.18 | 20.88 | 16.49 | 16.33 | 22.99 |
|  | 30 µM | 21.18 | 20.63 | 18.85 | 18.15 | 15.77 | 18.20 | 15.99 | 23.87 | 20.57 | 17.65 | 25.22 | 20.98 | 16.89 | 16.64 | 23.19 |
|  |  | 21.04 | 20.63 | 18.86 | 18.00 | 15.90 | 18.19 | 15.95 | 23.82 | 20.55 | 17.76 | 25.18 | 20.91 | 16.73 | 16.57 | 23.05 |
|  |  | 21.16 | 20.52 | 18.89 | 18.22 | 15.83 | 18.22 | 16.04 | 23.81 | 20.64 | 17.83 | 25.24 | 20.94 | 16.82 | 16.48 | 23.09 |

Table S6. The Ct values of melanoma-derived statin-sensitive MDA-MB-435 cell line

|  |  | Reference gene | | | | | | | | | | | | | | |
| --- | --- | --- | --- | --- | --- | --- | --- | --- | --- | --- | --- | --- | --- | --- | --- | --- |
|  |  | *ATP5F1* | *TFRC* | *YWHAZ* | *RPLP0* | *RPLP1* | *RPLP2* | *ACTB* | *HPRT1* | *B2M* | *RPS18* | *TBP* | *PGK1* | *PPIA* | *GAPDH* | *GUSB* |
| Concentration of atorvastatin | 0 µM | 20.89 | 20.79 | 19.63 | 18.03 | 15.63 | 17.91 | 15.21 | 22.17 | 20.06 | 16.89 | 23.91 | 19.92 | 17.51 | 16.12 | 23.95 |
|  |  | 20.85 | 20.97 | 19.65 | 18.14 | 15.59 | 17.79 | 15.14 | 22.17 | 20.07 | 16.74 | 23.94 | 19.96 | 17.28 | 16.15 | 23.89 |
|  |  | 20.75 | 20.92 | 19.57 | 17.99 | 15.52 | 17.83 | 15.16 | 22.06 | 20.05 | 16.70 | 23.96 | 19.90 | 17.27 | 16.06 | 23.90 |
|  | 0.1 µM | 20.95 | 21.00 | 19.51 | 18.07 | 15.56 | 17.90 | 15.36 | 22.23 | 20.11 | 16.97 | 24.17 | 19.96 | 17.28 | 16.08 | 24.01 |
|  |  | 20.72 | 21.04 | 19.46 | 17.97 | 15.46 | 17.77 | 15.19 | 22.16 | 19.99 | 16.75 | 24.12 | 19.90 | 17.32 | 16.19 | 23.88 |
|  |  | 20.92 | 20.91 | 19.52 | 18.01 | 15.53 | 17.79 | 15.13 | 21.99 | 20.06 | 16.89 | 24.22 | 19.92 | 17.34 | 16.21 | 23.87 |
|  | 0.3 µM | 20.94 | 20.96 | 19.53 | 17.96 | 15.53 | 17.78 | 15.10 | 22.10 | 20.07 | 16.79 | 24.10 | 19.84 | 17.43 | 16.20 | 23.93 |
|  |  | 20.82 | 20.99 | 19.56 | 17.89 | 15.38 | 17.80 | 15.12 | 22.00 | 19.99 | 16.72 | 24.04 | 19.81 | 17.20 | 16.26 | 23.90 |
|  |  | 20.78 | 20.85 | 19.46 | 17.94 | 15.48 | 17.83 | 15.08 | 22.04 | 19.94 | 16.85 | 24.06 | 19.81 | 17.32 | 16.16 | 23.94 |
|  | 1 µM | 20.87 | 20.98 | 19.35 | 18.07 | 15.59 | 17.73 | 15.17 | 22.09 | 20.06 | 16.98 | 24.02 | 19.83 | 17.06 | 16.16 | 23.98 |
|  |  | 20.85 | 20.82 | 19.29 | 18.05 | 15.45 | 17.67 | 15.14 | 22.11 | 20.08 | 16.72 | 24.00 | 19.60 | 17.22 | 16.22 | 23.90 |
|  |  | 20.89 | 20.97 | 19.54 | 18.02 | 15.44 | 17.71 | 15.13 | 22.01 | 19.99 | 16.82 | 24.08 | 19.79 | 17.27 | 16.21 | 23.85 |
|  | 3 µM | 20.69 | 20.73 | 19.17 | 17.91 | 15.42 | 17.77 | 15.19 | 22.05 | 20.25 | 16.68 | 24.00 | 19.76 | 17.05 | 16.03 | 23.94 |
|  |  | 20.90 | 20.72 | 19.30 | 18.03 | 15.31 | 17.76 | 15.29 | 22.06 | 20.04 | 16.83 | 24.05 | 19.82 | 17.11 | 16.31 | 24.03 |
|  |  | 20.92 | 20.76 | 19.23 | 18.03 | 15.53 | 17.90 | 15.32 | 22.05 | 20.01 | 16.92 | 24.14 | 19.80 | 17.04 | 16.10 | 23.90 |
|  | 10 µM | 20.93 | 19.95 | 19.45 | 17.96 | 15.85 | 17.81 | 15.81 | 21.86 | 20.07 | 17.15 | 23.99 | 19.77 | 17.25 | 16.25 | 24.04 |
|  |  | 21.07 | 20.13 | 19.06 | 18.31 | 15.58 | 17.86 | 15.98 | 21.95 | 20.17 | 16.91 | 24.10 | 19.69 | 17.24 | 16.11 | 23.98 |
|  |  | 20.78 | 20.00 | 19.18 | 17.98 | 15.53 | 17.79 | 15.73 | 21.92 | 20.13 | 16.88 | 24.10 | 19.56 | 17.07 | 15.99 | 24.11 |
|  | 30 µM | 21.21 | 19.93 | 19.09 | 17.81 | 15.55 | 17.70 | 15.81 | 21.72 | 19.97 | 16.94 | 23.88 | 19.65 | 17.13 | 16.21 | 23.89 |
|  |  | 21.07 | 19.89 | 19.05 | 17.99 | 15.44 | 17.81 | 15.89 | 21.74 | 19.94 | 16.91 | 23.98 | 19.74 | 17.09 | 16.29 | 24.00 |
|  |  | 21.08 | 20.01 | 19.16 | 17.75 | 15.63 | 17.81 | 15.75 | 21.81 | 19.99 | 17.08 | 24.08 | 19.75 | 17.32 | 16.30 | 23.98 |

Table S7. Gene expression stability ranking by five different algorithms on lung cancer cell lines.

|  | geNorm | |  | BestKeeper | |  | NormFinder | |  | RefFinder | |  | ΔCt method | |
| --- | --- | --- | --- | --- | --- | --- | --- | --- | --- | --- | --- | --- | --- | --- |
| Gene | M value | Rank |  | Std Dev [+/- CP] | Rank |  | Stability value | Rank |  | Geomean of ranking values | Rank |  | Mean SD | Rank |
| **HOP-92** |  |  |  |  |  |  |  |  |  |  |  |  |  |  |
| *ATP5F1* | 0.129 | 12 |  | 0.184 | 12 |  | 0.093 | 9 |  | 11.47 | 12 |  | 0.204 | 12 |
| *TFRC* | 0.148 | 13 |  | 0.228 | 13 |  | 0.070 | 1 |  | 13.00 | 13 |  | 0.240 | 13 |
| *YWHAZ* | 0.062 | 6 |  | 0.076 | 4 |  | 0.099 | 11 |  | 5.42 | 7 |  | 0.168 | 8 |
| *RPLP0* | 0.048 | 1 |  | 0.083 | 5 |  | 0.086 | 6 |  | 5.96 | 8 |  | 0.153 | 4 |
| *RPLP1* | 0.068 | 7 |  | 0.089 | 6 |  | 0.102 | 12 |  | 8.97 | 9 |  | 0.171 | 9 |
| *RPLP2* | 0.052 | 2 |  | 0.076 | 3 |  | 0.074 | 3 |  | 2.63 | 2 |  | 0.145 | 1 |
| *ACTB* | 0.186 | 15 |  | 0.320 | 15 |  | 0.138 | 14 |  | 15.00 | 15 |  | 0.319 | 15 |
| *HPRT1* | 0.166 | 14 |  | 0.252 | 14 |  | 0.171 | 15 |  | 14.00 | 14 |  | 0.265 | 14 |
| *B2M* | 0.056 | 3 |  | 0.070 | 1 |  | 0.073 | 2 |  | 3.94 | 5 |  | 0.154 | 5 |
| *RPS18* | 0.060 | 5 |  | 0.075 | 2 |  | 0.084 | 5 |  | 2.21 | 1 |  | 0.149 | 3 |
| *TBP* | 0.110 | 11 |  | 0.156 | 11 |  | 0.116 | 13 |  | 9.34 | 10 |  | 0.187 | 11 |
| *PGK1* | 0.082 | 9 |  | 0.125 | 10 |  | 0.096 | 10 |  | 9.92 | 11 |  | 0.184 | 10 |
| *PPIA* | 0.075 | 8 |  | 0.105 | 9 |  | 0.091 | 8 |  | 5.24 | 6 |  | 0.159 | 7 |
| *GAPDH* | 0.089 | 10 |  | 0.091 | 8 |  | 0.080 | 4 |  | 2.99 | 3 |  | 0.146 | 2 |
| *GUSB* | 0.059 | 4 |  | 0.091 | 7 |  | 0.090 | 7 |  | 3.64 | 4 |  | 0.154 | 6 |
|  |  |  |  |  |  |  |  |  |  |  |  |  |  |  |
| **NCI-H322M** |  |  |  |  |  |  |  |  |  |  |  |  |  |  |
| *ATP5F1* | 0.063 | 8 |  | 0.115 | 11 |  | 0.035 | 1 |  | 2.58 | 3 |  | 0.102 | 3 |
| *TFRC* | 0.059 | 7 |  | 0.101 | 8 |  | 0.070 | 5 |  | 4.95 | 5 |  | 0.108 | 6 |
| *YWHAZ* | 0.077 | 11 |  | 0.099 | 7 |  | 0.089 | 10 |  | 9.05 | 10 |  | 0.135 | 12 |
| *RPLP0* | 0.073 | 10 |  | 0.093 | 3 |  | 0.094 | 11 |  | 7.19 | 8 |  | 0.123 | 10 |
| *RPLP1* | 0.081 | 12 |  | 0.137 | 12 |  | 0.068 | 3 |  | 10.93 | 11 |  | 0.115 | 9 |
| *RPLP2* | 0.036 | 3 |  | 0.081 | 1 |  | 0.071 | 6 |  | 1.97 | 1 |  | 0.099 | 2 |
| *ACTB* | 0.133 | 15 |  | 0.353 | 15 |  | 0.167 | 14 |  | 15.00 | 15 |  | 0.366 | 15 |
| *HPRT1* | 0.097 | 14 |  | 0.180 | 14 |  | 0.044 | 2 |  | 12.16 | 13 |  | 0.149 | 14 |
| *B2M* | 0.069 | 9 |  | 0.102 | 9 |  | 0.095 | 12 |  | 11.59 | 12 |  | 0.131 | 11 |
| *RPS18* | 0.033 | 2 |  | 0.093 | 4 |  | 0.081 | 9 |  | 6.29 | 7 |  | 0.111 | 7 |
| *TBP* | 0.090 | 13 |  | 0.177 | 13 |  | 0.195 | 15 |  | 13.49 | 14 |  | 0.144 | 13 |
| *PGK1* | 0.051 | 5 |  | 0.097 | 5 |  | 0.069 | 4 |  | 2.11 | 2 |  | 0.103 | 5 |
| *PPIA* | 0.055 | 6 |  | 0.098 | 6 |  | 0.076 | 7 |  | 6.00 | 6 |  | 0.112 | 8 |
| *GAPDH* | 0.032 | 1 |  | 0.083 | 2 |  | 0.153 | 13 |  | 3.13 | 4 |  | 0.102 | 4 |
| *GUSB* | 0.047 | 4 |  | 0.103 | 10 |  | 0.080 | 8 |  | 8.65 | 9 |  | 0.096 | 1 |

Table S8. Gene expression stability ranking by five different algorithms on prostate cancer cell lines.

|  | geNorm | |  | BestKeeper | |  | NormFinder | |  | RefFinder | |  | ΔCt method | |
| --- | --- | --- | --- | --- | --- | --- | --- | --- | --- | --- | --- | --- | --- | --- |
| Gene | M value | Rank |  | Std Dev [+/- CP] | Rank |  | Stability value | Rank |  | Geomean of ranking values | Rank |  | Mean SD | Rank |
| **PC-3** |  |  |  |  |  |  |  |  |  |  |  |  |  |  |
| *ATP5F1* | 0.139 | 13 |  | 0.218 | 14 |  | 0.056 | 11 |  | 13.24 | 13 |  | 0.224 | 12 |
| *TFRC* | 0.126 | 12 |  | 0.218 | 13 |  | 0.047 | 9 |  | 11.98 | 12 |  | 0.214 | 11 |
| *YWHAZ* | 0.161 | 14 |  | 0.178 | 12 |  | 0.118 | 15 |  | 13.47 | 14 |  | 0.321 | 14 |
| *RPLP0* | 0.073 | 8 |  | 0.075 | 4 |  | 0.034 | 7 |  | 6.93 | 8 |  | 0.157 | 8 |
| *RPLP1* | 0.056 | 5 |  | 0.089 | 6 |  | 0.028 | 4 |  | 6.74 | 7 |  | 0.145 | 7 |
| *RPLP2* | 0.051 | 3 |  | 0.085 | 5 |  | 0.049 | 10 |  | 3.76 | 5 |  | 0.132 | 1 |
| *ACTB* | 0.092 | 10 |  | 0.142 | 11 |  | 0.027 | 3 |  | 9.19 | 9 |  | 0.169 | 10 |
| *HPRT1* | 0.187 | 15 |  | 0.352 | 15 |  | 0.103 | 14 |  | 15.00 | 15 |  | 0.352 | 15 |
| *B2M* | 0.050 | 2 |  | 0.053 | 1 |  | 0.031 | 5 |  | 3.31 | 3 |  | 0.145 | 6 |
| *RPS18* | 0.048 | 1 |  | 0.059 | 2 |  | 0.026 | 1 |  | 1.19 | 1 |  | 0.134 | 3 |
| *TBP* | 0.084 | 9 |  | 0.126 | 10 |  | 0.064 | 12 |  | 10.00 | 10 |  | 0.158 | 9 |
| *PGK1* | 0.111 | 11 |  | 0.125 | 9 |  | 0.101 | 13 |  | 10.69 | 11 |  | 0.231 | 13 |
| *PPIA* | 0.054 | 4 |  | 0.062 | 3 |  | 0.026 | 2 |  | 2.59 | 2 |  | 0.144 | 5 |
| *GAPDH* | 0.067 | 7 |  | 0.092 | 7 |  | 0.033 | 6 |  | 5.63 | 6 |  | 0.143 | 4 |
| *GUSB* | 0.061 | 6 |  | 0.092 | 8 |  | 0.037 | 8 |  | 3.46 | 4 |  | 0.133 | 2 |
|  |  |  |  |  |  |  |  |  |  |  |  |  |  |  |
| **DU-145** |  |  |  |  |  |  |  |  |  |  |  |  |  |  |
| *ATP5F1* | 0.098 | 15 |  | 0.134 | 15 |  | 0.033 | 13 |  | 15.00 | 15 |  | 0.171 | 15 |
| *TFRC* | 0.08 | 13 |  | 0.101 | 14 |  | 0.026 | 6 |  | 13.24 | 13 |  | 0.130 | 13 |
| *YWHAZ* | 0.064 | 11 |  | 0.071 | 8 |  | 0.021 | 4 |  | 5.83 | 7 |  | 0.095 | 11 |
| *RPLP0* | 0.041 | 5 |  | 0.078 | 10 |  | 0.028 | 9 |  | 9.46 | 10 |  | 0.084 | 7 |
| *RPLP1* | 0.026 | 1 |  | 0.079 | 11 |  | 0.028 | 8 |  | 11.00 | 11 |  | 0.074 | 2 |
| *RPLP2* | 0.031 | 3 |  | 0.059 | 3 |  | 0.022 | 5 |  | 1.86 | 2 |  | 0.076 | 3 |
| *ACTB* | 0.047 | 7 |  | 0.057 | 2 |  | 0.019 | 3 |  | 4.92 | 5 |  | 0.084 | 6 |
| *HPRT1* | 0.05 | 8 |  | 0.061 | 4 |  | 0.027 | 7 |  | 4.47 | 4 |  | 0.086 | 8 |
| *B2M* | 0.052 | 9 |  | 0.071 | 7 |  | 0.015 | 2 |  | 7.94 | 8 |  | 0.088 | 9 |
| *RPS18* | 0.071 | 12 |  | 0.090 | 13 |  | 0.045 | 15 |  | 13.74 | 14 |  | 0.126 | 12 |
| *TBP* | 0.046 | 6 |  | 0.067 | 6 |  | 0.039 | 14 |  | 5.73 | 6 |  | 0.083 | 5 |
| *PGK1* | 0.087 | 14 |  | 0.081 | 12 |  | 0.030 | 11 |  | 12.00 | 12 |  | 0.131 | 14 |
| *PPIA* | 0.034 | 4 |  | 0.052 | 1 |  | 0.009 | 1 |  | 1.00 | 1 |  | 0.069 | 1 |
| *GAPDH* | 0.059 | 10 |  | 0.075 | 9 |  | 0.030 | 12 |  | 8.71 | 9 |  | 0.093 | 10 |
| *GUSB* | 0.028 | 2 |  | 0.066 | 5 |  | 0.030 | 10 |  | 3.41 | 3 |  | 0.081 | 4 |

Table S9. Gene expression stability ranking by five different algorithms on melanoma cell lines.

|  | geNorm | |  | BestKeeper | |  | NormFinder | |  | RefFinder | |  | ΔCt method | |
| --- | --- | --- | --- | --- | --- | --- | --- | --- | --- | --- | --- | --- | --- | --- |
| Gene | M value | Rank |  | Std Dev [+/- CP] | Rank |  | Stability value | Rank |  | Geomean of ranking values | Rank |  | Mean SD | Rank |
| **SK-MEL-5** |  |  |  |  |  |  |  |  |  |  |  |  |  |  |
| *ATP5F1* | 0.031 | 3 |  | 0.065 | 6 |  | 0.034 | 2 |  | 4.43 | 4 |  | 0.087 | 3 |
| *TFRC* | 0.051 | 7 |  | 0.052 | 2 |  | 0.051 | 7 |  | 4.90 | 5 |  | 0.098 | 8 |
| *YWHAZ* | 0.029 | 2 |  | 0.049 | 1 |  | 0.041 | 4 |  | 1.19 | 1 |  | 0.081 | 1 |
| *RPLP0* | 0.038 | 4 |  | 0.072 | 8 |  | 0.039 | 3 |  | 3.83 | 3 |  | 0.089 | 4 |
| *RPLP1* | 0.043 | 5 |  | 0.062 | 5 |  | 0.067 | 10 |  | 6.40 | 6 |  | 0.092 | 5 |
| *RPLP2* | 0.027 | 1 |  | 0.057 | 4 |  | 0.033 | 1 |  | 1.68 | 2 |  | 0.082 | 2 |
| *ACTB* | 0.117 | 15 |  | 0.259 | 15 |  | 0.164 | 15 |  | 15.00 | 15 |  | 0.297 | 15 |
| *HPRT1* | 0.071 | 11 |  | 0.100 | 12 |  | 0.042 | 5 |  | 11.72 | 12 |  | 0.124 | 11 |
| *B2M* | 0.09 | 14 |  | 0.066 | 7 |  | 0.048 | 6 |  | 11.33 | 11 |  | 0.149 | 14 |
| *RPS18* | 0.048 | 6 |  | 0.072 | 9 |  | 0.053 | 8 |  | 9.49 | 10 |  | 0.096 | 7 |
| *TBP* | 0.077 | 12 |  | 0.118 | 14 |  | 0.079 | 12 |  | 12.96 | 14 |  | 0.125 | 12 |
| *PGK1* | 0.055 | 8 |  | 0.054 | 3 |  | 0.065 | 9 |  | 6.59 | 7 |  | 0.106 | 10 |
| *PPIA* | 0.083 | 13 |  | 0.104 | 13 |  | 0.096 | 14 |  | 12.47 | 13 |  | 0.134 | 13 |
| *GAPDH* | 0.058 | 9 |  | 0.078 | 11 |  | 0.082 | 13 |  | 7.45 | 9 |  | 0.106 | 9 |
| *GUSB* | 0.061 | 10 |  | 0.073 | 10 |  | 0.067 | 11 |  | 6.89 | 8 |  | 0.096 | 6 |
|  |  |  |  |  |  |  |  |  |  |  |  |  |  |  |
| **MDA-MB-435** |  |  |  |  |  |  |  |  |  |  |  |  |  |  |
| *ATP5F1* | 0.093 | 9 |  | 0.095 | 9 |  | 0.090 | 6 |  | 10.67 | 11 |  | 0.182 | 11 |
| *TFRC* | 0.196 | 15 |  | 0.371 | 15 |  | 0.445 | 15 |  | 15.00 | 15 |  | 0.464 | 15 |
| *YWHAZ* | 0.125 | 13 |  | 0.174 | 13 |  | 0.119 | 9 |  | 13.00 | 13 |  | 0.219 | 13 |
| *RPLP0* | 0.063 | 4 |  | 0.076 | 6 |  | 0.070 | 3 |  | 6.93 | 8 |  | 0.152 | 5 |
| *RPLP1* | 0.068 | 5 |  | 0.077 | 7 |  | 0.126 | 10 |  | 6.19 | 6 |  | 0.152 | 4 |
| *RPLP2* | 0.054 | 3 |  | 0.048 | 1 |  | 0.068 | 2 |  | 1.00 | 1 |  | 0.139 | 1 |
| *ACTB* | 0.155 | 14 |  | 0.263 | 14 |  | 0.237 | 14 |  | 14.00 | 14 |  | 0.368 | 14 |
| *HPRT1* | 0.113 | 12 |  | 0.107 | 12 |  | 0.145 | 13 |  | 11.22 | 12 |  | 0.183 | 12 |
| *B2M* | 0.053 | 2 |  | 0.056 | 3 |  | 0.063 | 1 |  | 3.41 | 3 |  | 0.143 | 2 |
| *RPS18* | 0.077 | 6 |  | 0.097 | 10 |  | 0.143 | 12 |  | 9.01 | 9 |  | 0.163 | 10 |
| *TBP* | 0.082 | 7 |  | 0.070 | 4 |  | 0.106 | 7 |  | 4.00 | 4 |  | 0.155 | 6 |
| *PGK1* | 0.105 | 11 |  | 0.084 | 8 |  | 0.110 | 8 |  | 5.18 | 5 |  | 0.161 | 8 |
| *PPIA* | 0.1 | 10 |  | 0.103 | 11 |  | 0.074 | 5 |  | 9.95 | 10 |  | 0.163 | 9 |
| *GAPDH* | 0.087 | 8 |  | 0.073 | 5 |  | 0.131 | 11 |  | 6.65 | 7 |  | 0.158 | 7 |
| *GUSB* | 0.053 | 1 |  | 0.053 | 2 |  | 0.072 | 4 |  | 1.86 | 2 |  | 0.143 | 3 |
