# Supplementary material for: Expression of housekeeping genes varies depending on mevalonate pathway inhibition in cancer cells
Source: Heliyon. 2023 Jul 8;9(7):e18017. doi: 10.1016/j.heliyon.2023.e18017 (PMC10368838; doi:10.1016/j.heliyon.2023.e18017)
Supplement: Multimedia component 2 [file mmc2.pptx]

## Slide 1
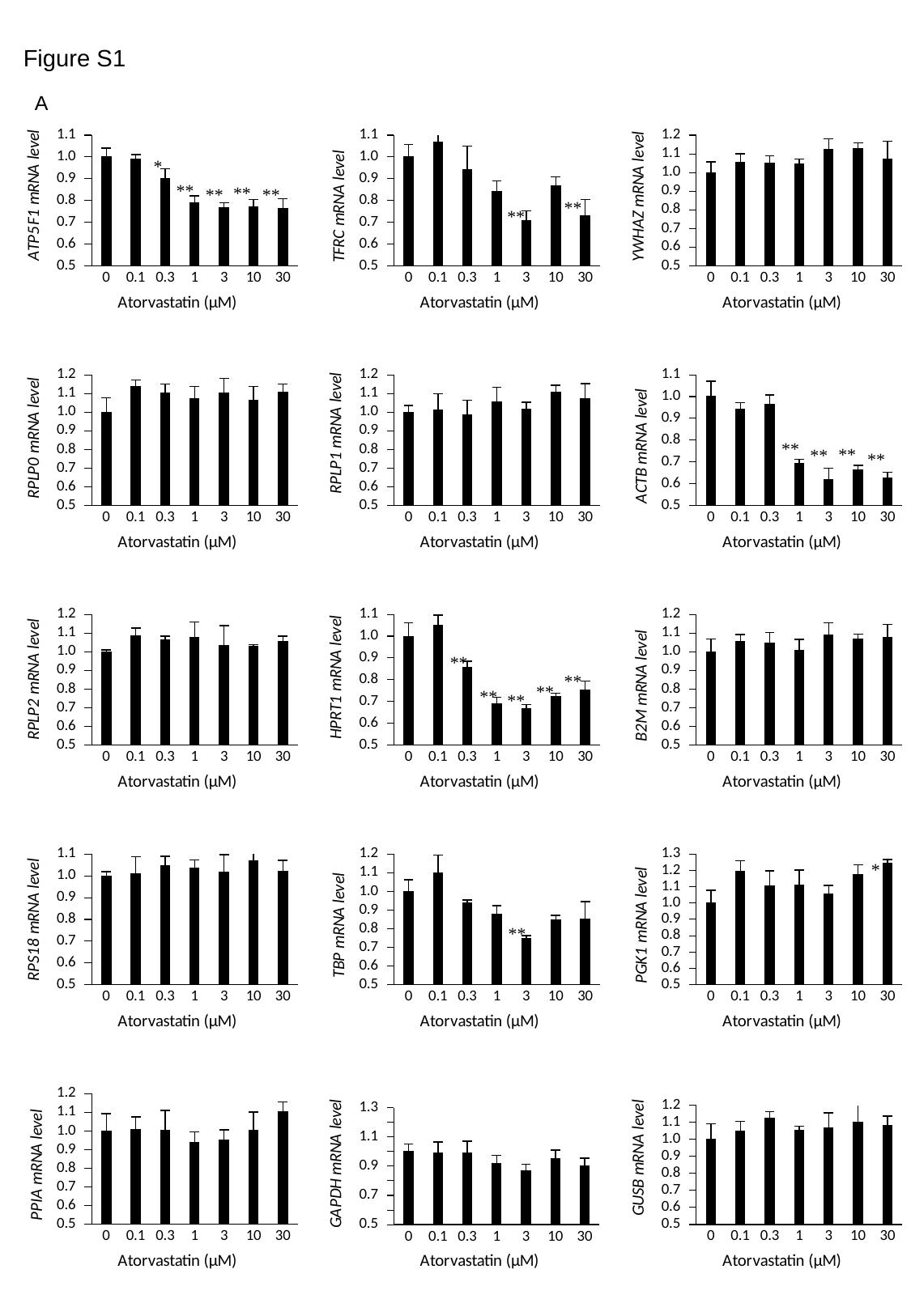

Figure S1
A
### Chart
| Category | |
|---|---|
| 0 | 1.0 |
| 0.1 | 0.9889220043981339 |
| 0.3 | 0.8997635084930686 |
| 1 | 0.791549706455427 |
| 3 | 0.7667050837579024 |
| 10 | 0.7708864469582865 |
| 30 | 0.7655013522544089 |
### Chart
| Category | |
|---|---|
| 0 | 1.0 |
| 0.1 | 1.06756185058188 |
| 0.3 | 0.9434859754777841 |
| 1 | 0.8412228001949016 |
| 3 | 0.7077238460475748 |
| 10 | 0.8681714218284405 |
| 30 | 0.732067014540446 |
### Chart
| Category | |
|---|---|
| 0 | 1.0 |
| 0.1 | 1.0552179873221306 |
| 0.3 | 1.0508606172645678 |
| 1 | 1.04668249039647 |
| 3 | 1.1226103474035856 |
| 10 | 1.1294712898271808 |
| 30 | 1.0726820627920624 |*
**
**
**
**
**
**
### Chart
| Category | |
|---|---|
| 0 | 1.0 |
| 0.1 | 1.1380712191868978 |
| 0.3 | 1.1039718404659515 |
| 1 | 1.072154056048383 |
| 3 | 1.1011739742102558 |
| 10 | 1.0650559493393887 |
| 30 | 1.1069552244533278 |
### Chart
| Category | |
|---|---|
| 0 | 1.0 |
| 0.1 | 1.0103952524160142 |
| 0.3 | 0.9864504648983076 |
| 1 | 1.0561076935794225 |
| 3 | 1.0165405065688464 |
| 10 | 1.1061489694327111 |
| 30 | 1.0737483861438386 |
### Chart
| Category | |
|---|---|
| 0 | 1.0000000000000002 |
| 0.1 | 0.9425674598782977 |
| 0.3 | 0.964749183219835 |
| 1 | 0.6918156533571352 |
| 3 | 0.620546189370228 |
| 10 | 0.665174951461596 |
| 30 | 0.6256148970898794 |**
**
**
**
### Chart
| Category | |
|---|---|
| 0 | 1.0 |
| 0.1 | 1.0856393666394502 |
| 0.3 | 1.0646839136715114 |
| 1 | 1.0751313202706474 |
| 3 | 1.0349941275975219 |
| 10 | 1.0280502623303522 |
| 30 | 1.0539443177499466 |
### Chart
| Category | |
|---|---|
| 0 | 1.0 |
| 0.1 | 1.0510449564674291 |
| 0.3 | 0.8565901128355274 |
| 1 | 0.6910262049243471 |
| 3 | 0.6683762876738634 |
| 10 | 0.7250412963021379 |
| 30 | 0.7512027722156628 |
### Chart
| Category | |
|---|---|
| 0 | 1.0 |
| 0.1 | 1.055860859537481 |
| 0.3 | 1.0460782318216157 |
| 1 | 1.0085017948943389 |
| 3 | 1.091005018447063 |
| 10 | 1.0694514447774903 |
| 30 | 1.0784034796226702 |**
**
**
**
### Chart
| Category | |
|---|---|
| 0 | 1.0 |
| 0.1 | 1.0081732136163333 |
| 0.3 | 1.0480833722205898 |
| 1 | 1.0366821277185243 |
| 3 | 1.016802660740353 |
| 10 | 1.0684791348028713 |
| 30 | 1.0209326749480365 |
### Chart
| Category | |
|---|---|
| 0 | 1.0 |
| 0.1 | 1.0989420330555435 |
| 0.3 | 0.9388263809916856 |
| 1 | 0.8772928664543139 |
| 3 | 0.7500074795087691 |
| 10 | 0.8458699400343322 |
| 30 | 0.8521516781422891 |
### Chart
| Category | |
|---|---|
| 0 | 1.0 |
| 0.1 | 1.1956569224945477 |
| 0.3 | 1.1039815355924747 |
| 1 | 1.110146389403208 |
| 3 | 1.0554893643625043 |
| 10 | 1.17496638658986 |
| 30 | 1.2424575014800574 |*
**
### Chart
| Category | |
|---|---|
| 0 | 1.0 |
| 0.1 | 1.0081973276671883 |
| 0.3 | 1.00468492422865 |
| 1 | 0.9402252383480229 |
| 3 | 0.9497830500286489 |
| 10 | 1.002194941231322 |
| 30 | 1.1045480559256458 |
### Chart
| Category | |
|---|---|
| 0 | 1.0 |
| 0.1 | 1.0478536826481768 |
| 0.3 | 1.1257640577590833 |
| 1 | 1.0510727136801288 |
| 3 | 1.0657462784660379 |
| 10 | 1.0994750761285095 |
| 30 | 1.083306359742806 |
### Chart
| Category | |
|---|---|
| 0 | 1.0 |
| 0.1 | 0.9912841684174597 |
| 0.3 | 0.9917701586592331 |
| 1 | 0.9205618011619143 |
| 3 | 0.8706220791986469 |
| 10 | 0.9499667392090217 |
| 30 | 0.9049812716513417 |

## Slide 2
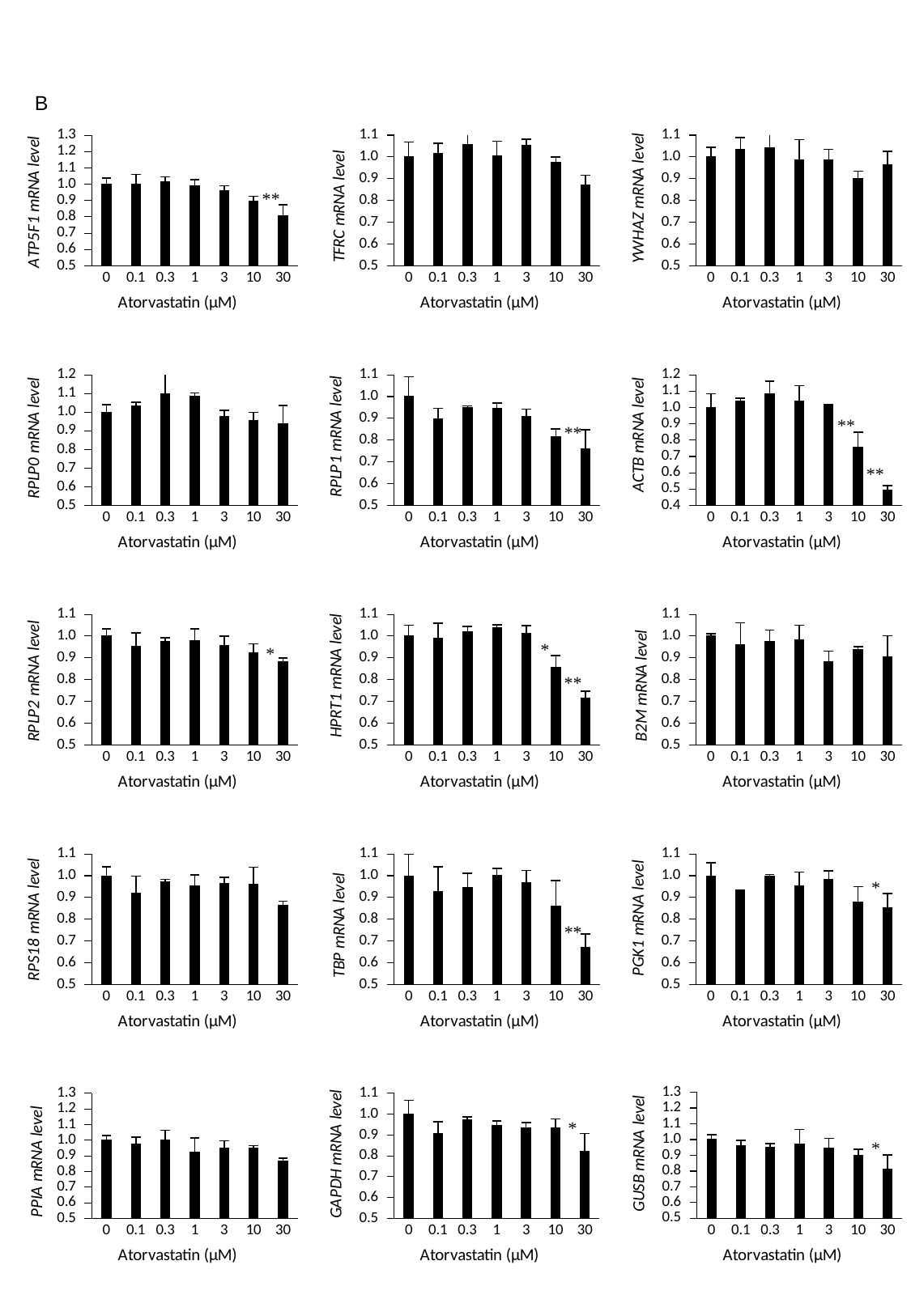

B
### Chart
| Category | |
|---|---|
| 0 | 1.0 |
| 0.1 | 1.0022555600554723 |
| 0.3 | 1.0138921736689959 |
| 1 | 0.9906296062328911 |
| 3 | 0.9594282589307768 |
| 10 | 0.8947187338586701 |
| 30 | 0.8054338562756298 |
### Chart
| Category | |
|---|---|
| 0 | 1.0 |
| 0.1 | 1.0156489149607308 |
| 0.3 | 1.057773500165359 |
| 1 | 1.006111124607184 |
| 3 | 1.0526250440068374 |
| 10 | 0.9762401766263186 |
| 30 | 0.8717921152343541 |
### Chart
| Category | |
|---|---|
| 0 | 1.0 |
| 0.1 | 1.0344740420569953 |
| 0.3 | 1.0415830037063027 |
| 1 | 0.9876095516170255 |
| 3 | 0.9874655487613878 |
| 10 | 0.9025760122713548 |
| 30 | 0.9648634664559371 |**
### Chart
| Category | |
|---|---|
| 0 | 1.0 |
| 0.1 | 1.0319108005384263 |
| 0.3 | 1.0995431261723436 |
| 1 | 1.0843965652824743 |
| 3 | 0.9783545256907425 |
| 10 | 0.9554837787149623 |
| 30 | 0.9397224816121249 |
### Chart
| Category | |
|---|---|
| 0 | 1.0 |
| 0.1 | 0.898870718801144 |
| 0.3 | 0.9504873402595845 |
| 1 | 0.9469397160304807 |
| 3 | 0.9106325256510622 |
| 10 | 0.8170072748416061 |
| 30 | 0.7608314905356149 |
### Chart
| Category | |
|---|---|
| 0 | 1.0 |
| 0.1 | 1.040768080252195 |
| 0.3 | 1.0840323686271478 |
| 1 | 1.038364520590477 |
| 3 | 1.0137186512565477 |
| 10 | 0.7551791684592284 |
| 30 | 0.494311602019957 |**
**
**
### Chart
| Category | |
|---|---|
| 0 | 1.0 |
| 0.1 | 0.9540474128575515 |
| 0.3 | 0.9745830978953299 |
| 1 | 0.9797308399865611 |
| 3 | 0.9565748992436304 |
| 10 | 0.9225178920904256 |
| 30 | 0.8814131969302189 |
### Chart
| Category | |
|---|---|
| 0 | 1.0 |
| 0.1 | 0.9891082401896744 |
| 0.3 | 1.0208997615125097 |
| 1 | 1.037379443591169 |
| 3 | 1.0146517569198243 |
| 10 | 0.8579607639797894 |
| 30 | 0.7173089474750688 |
### Chart
| Category | |
|---|---|
| 0 | 1.0 |
| 0.1 | 0.962794023615547 |
| 0.3 | 0.9753210598773516 |
| 1 | 0.9818158027230632 |
| 3 | 0.8816939974730692 |
| 10 | 0.9378408017587915 |
| 30 | 0.9039407673758317 |*
*
**
### Chart
| Category | |
|---|---|
| 0 | 1.0 |
| 0.1 | 0.9218881625592531 |
| 0.3 | 0.9709528290666039 |
| 1 | 0.9540320091168372 |
| 3 | 0.9660436956622857 |
| 10 | 0.9604601778619243 |
| 30 | 0.8652838220270307 |
### Chart
| Category | |
|---|---|
| 0 | 1.0000000000000002 |
| 0.1 | 0.9287679230453504 |
| 0.3 | 0.9463327169000036 |
| 1 | 1.003889203840083 |
| 3 | 0.9698028166699041 |
| 10 | 0.862148364718435 |
| 30 | 0.6725792005669438 |
### Chart
| Category | |
|---|---|
| 0 | 1.0 |
| 0.1 | 0.9309983591863409 |
| 0.3 | 0.9993648336431221 |
| 1 | 0.9521401672692732 |
| 3 | 0.9836009059212981 |
| 10 | 0.8776339572998292 |
| 30 | 0.8541391674256543 |*
**
### Chart
| Category | |
|---|---|
| 0 | 1.0 |
| 0.1 | 0.9641006346282953 |
| 0.3 | 0.9526210875742135 |
| 1 | 0.9737956516136781 |
| 3 | 0.9473938340057325 |
| 10 | 0.8979470864467854 |
| 30 | 0.8124173610020743 |
### Chart
| Category | |
|---|---|
| 0 | 1.0 |
| 0.1 | 0.9776498427990784 |
| 0.3 | 0.9994092317323382 |
| 1 | 0.9253849190932392 |
| 3 | 0.947703067747542 |
| 10 | 0.9502422720576108 |
| 30 | 0.8665911994985946 |
### Chart
| Category | |
|---|---|
| 0 | 1.0000000000000002 |
| 0.1 | 0.9078086460297046 |
| 0.3 | 0.9730318368053404 |
| 1 | 0.9453864393466332 |
| 3 | 0.9342578744327144 |
| 10 | 0.9324909125742442 |
| 30 | 0.8210278672220613 |*
*

## Slide 3
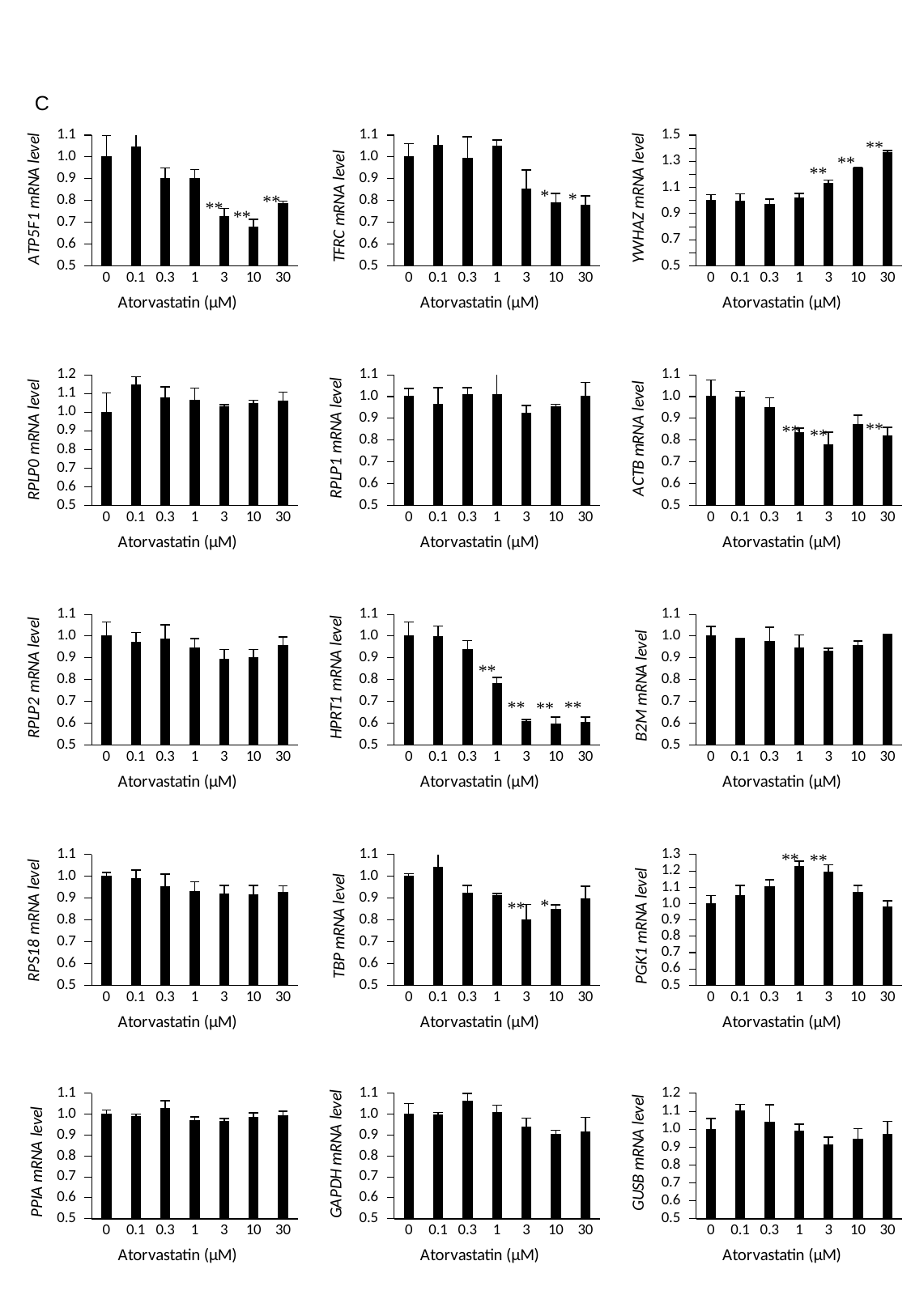

C
### Chart
| Category | |
|---|---|
| 0 | 1.0 |
| 0.1 | 1.0468107187005702 |
| 0.3 | 0.90310073547505 |
| 1 | 0.9023636241704832 |
| 3 | 0.725368260552039 |
| 10 | 0.6795601970495052 |
| 30 | 0.784973506669071 |
### Chart
| Category | |
|---|---|
| 0 | 0.9999999999999999 |
| 0.1 | 1.05507957876262 |
| 0.3 | 0.9936889880706534 |
| 1 | 1.0495731533120767 |
| 3 | 0.8540812264981262 |
| 10 | 0.7888395450139875 |
| 30 | 0.7800623143075263 |
### Chart
| Category | |
|---|---|
| 0 | 1.0 |
| 0.1 | 0.9937904690530982 |
| 0.3 | 0.9714941543436598 |
| 1 | 1.0182093457047223 |
| 3 | 1.1303609705438722 |
| 10 | 1.2506670282816497 |
| 30 | 1.3694603688422091 |**
**
**
*
*
**
**
**
### Chart
| Category | |
|---|---|
| 0 | 1.0 |
| 0.1 | 1.144605493984804 |
| 0.3 | 1.077895750203876 |
| 1 | 1.0645617173559703 |
| 3 | 1.029339635058743 |
| 10 | 1.0479872296195667 |
| 30 | 1.057843164725812 |
### Chart
| Category | |
|---|---|
| 0 | 1.0 |
| 0.1 | 0.9659860048899276 |
| 0.3 | 1.009692270796019 |
| 1 | 1.0103222137668657 |
| 3 | 0.9243771652961356 |
| 10 | 0.9552923760473756 |
| 30 | 1.0022833713757715 |
### Chart
| Category | |
|---|---|
| 0 | 1.0 |
| 0.1 | 0.997330581398094 |
| 0.3 | 0.9493563230102625 |
| 1 | 0.8345238808251332 |
| 3 | 0.7793211699783913 |
| 10 | 0.8708188581291231 |
| 30 | 0.8214824912055291 |**
**
**
### Chart
| Category | |
|---|---|
| 0 | 1.0 |
| 0.1 | 0.9710710212998727 |
| 0.3 | 0.9885987187919062 |
| 1 | 0.9446835366632497 |
| 3 | 0.8938594298993259 |
| 10 | 0.9022425064536906 |
| 30 | 0.956589396730437 |
### Chart
| Category | |
|---|---|
| 0 | 1.0 |
| 0.1 | 0.9970049919325362 |
| 0.3 | 0.9387115359459305 |
| 1 | 0.7825171463054921 |
| 3 | 0.6084763678619886 |
| 10 | 0.5956995319053552 |
| 30 | 0.603676880405655 |
### Chart
| Category | |
|---|---|
| 0 | 1.0 |
| 0.1 | 0.9837075092565176 |
| 0.3 | 0.9766258551249756 |
| 1 | 0.945553984355624 |
| 3 | 0.9328189743470486 |
| 10 | 0.9583290999644211 |
| 30 | 1.0074473301546485 |**
**
**
**
**
**
### Chart
| Category | |
|---|---|
| 0 | 1.0 |
| 0.1 | 0.9902058276599354 |
| 0.3 | 0.954934225198461 |
| 1 | 0.9323613793444175 |
| 3 | 0.920392087316151 |
| 10 | 0.91594978936726 |
| 30 | 0.9283664147596019 |
### Chart
| Category | |
|---|---|
| 0 | 1.0 |
| 0.1 | 1.0407187113585805 |
| 0.3 | 0.9226477658935979 |
| 1 | 0.9132587971707727 |
| 3 | 0.7992017301314549 |
| 10 | 0.8509691164778692 |
| 30 | 0.8958461349156366 |
### Chart
| Category | |
|---|---|
| 0 | 1.0 |
| 0.1 | 1.05060636990374 |
| 0.3 | 1.1029774527211733 |
| 1 | 1.2279029816410596 |
| 3 | 1.1936510417056627 |
| 10 | 1.0687594773591496 |
| 30 | 0.9817723768977024 |*
**
### Chart
| Category | |
|---|---|
| 0 | 1.0000000000000002 |
| 0.1 | 0.9906871072625355 |
| 0.3 | 1.027553336292869 |
| 1 | 0.9718161493747086 |
| 3 | 0.9656579965675863 |
| 10 | 0.9849682772194174 |
| 30 | 0.994426827892467 |
### Chart
| Category | |
|---|---|
| 0 | 1.0 |
| 0.1 | 0.9974832074674094 |
| 0.3 | 1.063603611421749 |
| 1 | 1.0071202179002448 |
| 3 | 0.9370445208383874 |
| 10 | 0.9030748562025374 |
| 30 | 0.916803051550133 |
### Chart
| Category | |
|---|---|
| 0 | 1.0 |
| 0.1 | 1.1005790633659138 |
| 0.3 | 1.037586346504728 |
| 1 | 0.989958456067527 |
| 3 | 0.9137491130713006 |
| 10 | 0.9456605969787879 |
| 30 | 0.9711778719924693 |

## Slide 4
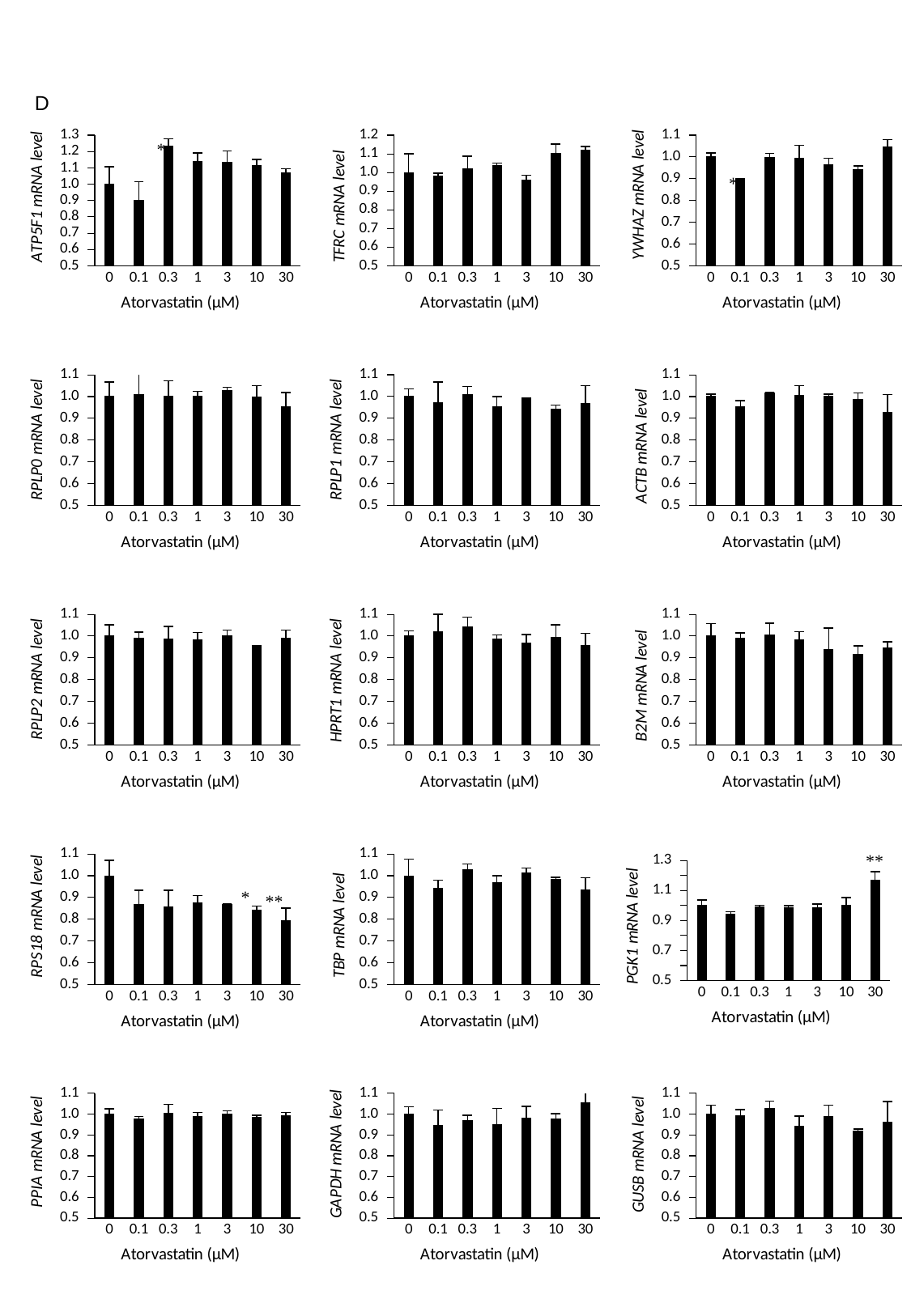

D
### Chart
| Category | |
|---|---|
| 0 | 1.0 |
| 0.1 | 0.9023329042144148 |
| 0.3 | 1.2326169391357482 |
| 1 | 1.1383205462813162 |
| 3 | 1.133704577495638 |
| 10 | 1.115209238776994 |
| 30 | 1.0678418014749331 |
### Chart
| Category | |
|---|---|
| 0 | 1.0 |
| 0.1 | 0.9827094900880486 |
| 0.3 | 1.0189318218021377 |
| 1 | 1.0363468403451213 |
| 3 | 0.9603043614759258 |
| 10 | 1.1034732671845968 |
| 30 | 1.1192383977711964 |
### Chart
| Category | |
|---|---|
| 0 | 1.0 |
| 0.1 | 0.897932549345525 |
| 0.3 | 0.9970720276918511 |
| 1 | 0.994298029452121 |
| 3 | 0.9631750647293362 |
| 10 | 0.9434949221593615 |
| 30 | 1.0452632682115441 |*
*
### Chart
| Category | |
|---|---|
| 0 | 1.0 |
| 0.1 | 0.9726030817997916 |
| 0.3 | 1.0073844214775114 |
| 1 | 0.9541274742314458 |
| 3 | 0.985684625136772 |
| 10 | 0.9433358834785549 |
| 30 | 0.9691397498884484 |
### Chart
| Category | |
|---|---|
| 0 | 1.0 |
| 0.1 | 1.0081879454522824 |
| 0.3 | 1.0009969436995443 |
| 1 | 1.0006574201708396 |
| 3 | 1.0288382448622742 |
| 10 | 0.9963573258646007 |
| 30 | 0.9524412680531443 |
### Chart
| Category | |
|---|---|
| 0 | 1.0 |
| 0.1 | 0.953177495586447 |
| 0.3 | 1.0148814055749402 |
| 1 | 1.0067850828633762 |
| 3 | 1.0017919057007674 |
| 10 | 0.9854351023617712 |
| 30 | 0.9263618684393023 |
### Chart
| Category | |
|---|---|
| 0 | 1.0 |
| 0.1 | 0.9893714481146364 |
| 0.3 | 0.9872487622466579 |
| 1 | 0.9844395436766762 |
| 3 | 1.0018259959282805 |
| 10 | 0.953073969847959 |
| 30 | 0.989442611521636 |
### Chart
| Category | |
|---|---|
| 0 | 1.0 |
| 0.1 | 1.0216869571491747 |
| 0.3 | 1.0409519351557002 |
| 1 | 0.9850512134419932 |
| 3 | 0.969074358916747 |
| 10 | 0.9954728719269509 |
| 30 | 0.9561797236744395 |
### Chart
| Category | |
|---|---|
| 0 | 1.0 |
| 0.1 | 0.9894987950114613 |
| 0.3 | 1.0073718553243005 |
| 1 | 0.9834514389112422 |
| 3 | 0.9381609290113125 |
| 10 | 0.9163574075931061 |
| 30 | 0.9448421734611089 |
### Chart
| Category | |
|---|---|
| 0 | 1.0 |
| 0.1 | 0.8672487048484793 |
| 0.3 | 0.8566276972923651 |
| 1 | 0.8772533154348751 |
| 3 | 0.8677776379597315 |
| 10 | 0.8427957020885394 |
| 30 | 0.7953813615009309 |
### Chart
| Category | |
|---|---|
| 0 | 1.0 |
| 0.1 | 0.9416112816034801 |
| 0.3 | 1.0284208819369824 |
| 1 | 0.9702436277809848 |
| 3 | 1.013495390031884 |
| 10 | 0.9831910040072042 |
| 30 | 0.9358788826415086 |
### Chart
| Category | |
|---|---|
| 0 | 1.0 |
| 0.1 | 0.9411097246623031 |
| 0.3 | 0.9911750502367349 |
| 1 | 0.9878901092550784 |
| 3 | 0.9848137487284179 |
| 10 | 0.999746756579673 |
| 30 | 1.1670488406291275 |**
*
**
### Chart
| Category | |
|---|---|
| 0 | 1.0 |
| 0.1 | 0.9765761023474852 |
| 0.3 | 1.0056350243498386 |
| 1 | 0.9889425593249123 |
| 3 | 0.9988813665456755 |
| 10 | 0.9839568464463969 |
| 30 | 0.9930655220491378 |
### Chart
| Category | |
|---|---|
| 0 | 1.0 |
| 0.1 | 0.9466454697138541 |
| 0.3 | 0.969804072278869 |
| 1 | 0.9478360056009537 |
| 3 | 0.9791201771292393 |
| 10 | 0.9772509711729668 |
| 30 | 1.0543643763486015 |
### Chart
| Category | |
|---|---|
| 0 | 1.0 |
| 0.1 | 0.9913576748340519 |
| 0.3 | 1.026297024988114 |
| 1 | 0.9400519520819607 |
| 3 | 0.9904524487014892 |
| 10 | 0.9176247268807578 |
| 30 | 0.9608741352666129 |

## Slide 5
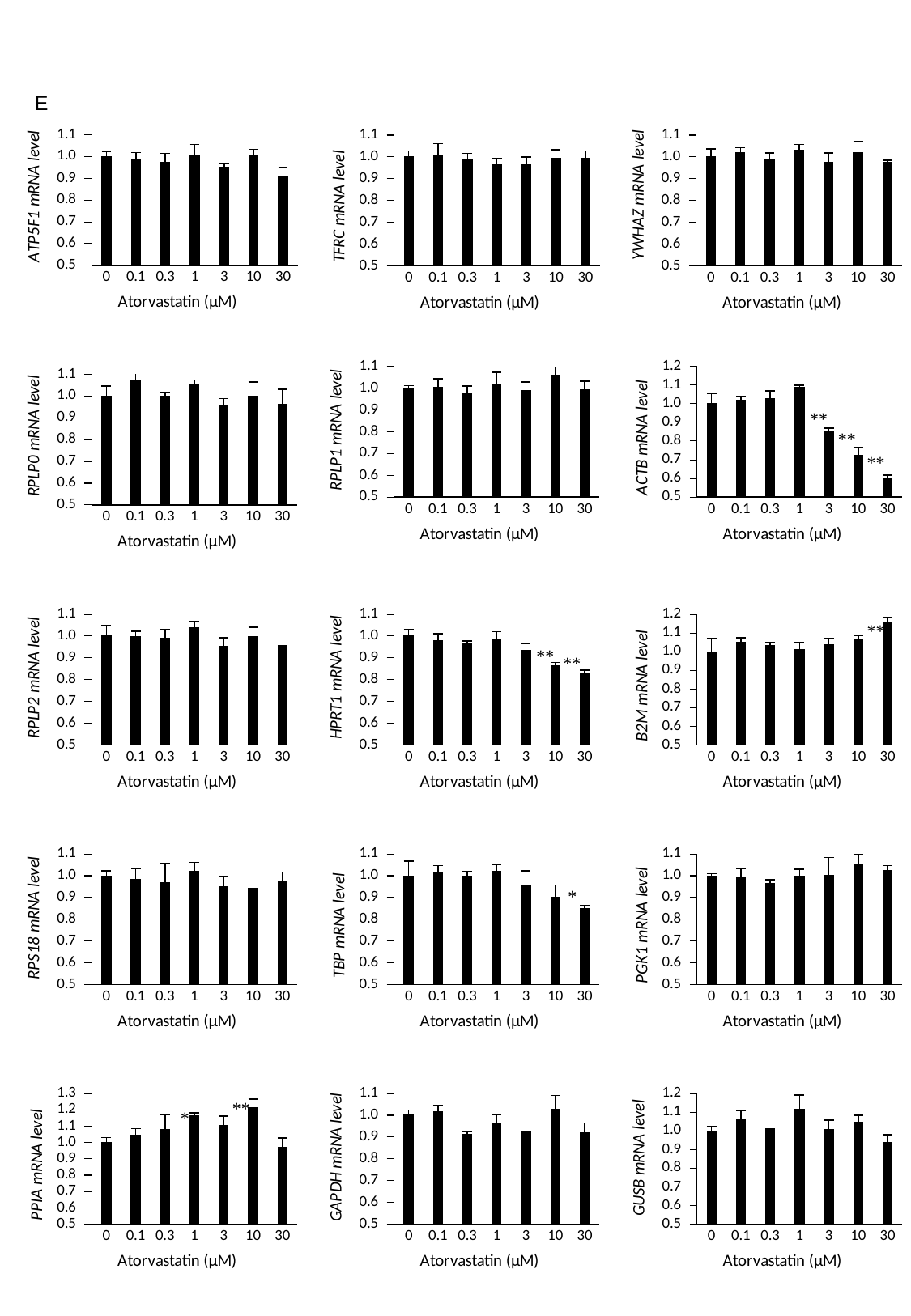

E
### Chart
| Category | |
|---|---|
| 0 | 1.0 |
| 0.1 | 0.9864511127955145 |
| 0.3 | 0.9742088605809595 |
| 1 | 1.0036497552508643 |
| 3 | 0.9511123398402744 |
| 10 | 1.005045008028112 |
| 30 | 0.9108551494962978 |
### Chart
| Category | |
|---|---|
| 0 | 1.0 |
| 0.1 | 1.00923917694078 |
| 0.3 | 0.9889242372975208 |
| 1 | 0.9647491399722136 |
| 3 | 0.9643033994446534 |
| 10 | 0.9936434102348337 |
| 30 | 0.992938558927387 |
### Chart
| Category | |
|---|---|
| 0 | 1.0 |
| 0.1 | 1.019151938968962 |
| 0.3 | 0.9911506230373294 |
| 1 | 1.0328733057354913 |
| 3 | 0.9758234521171908 |
| 10 | 1.0212165877050308 |
| 30 | 0.9743462945578694 |
### Chart
| Category | |
|---|---|
| 0 | 1.0 |
| 0.1 | 1.0043295359006261 |
| 0.3 | 0.9756037414178734 |
| 1 | 1.0201463016293457 |
| 3 | 0.9880209304822168 |
| 10 | 1.0611346837704716 |
| 30 | 0.9917587902305031 |
### Chart
| Category | |
|---|---|
| 0 | 1.0 |
| 0.1 | 1.0203161878854348 |
| 0.3 | 1.0275968939199605 |
| 1 | 1.0883101955192365 |
| 3 | 0.8523160321238663 |
| 10 | 0.7252873439878836 |
| 30 | 0.6005213490868478 |
### Chart
| Category | |
|---|---|
| 0 | 1.0 |
| 0.1 | 1.070977199662672 |
| 0.3 | 0.9985635727124054 |
| 1 | 1.0539076155675773 |
| 3 | 0.9558842460607688 |
| 10 | 1.000664508303923 |
| 30 | 0.9624458355278808 |**
**
**
### Chart
| Category | |
|---|---|
| 0 | 1.0 |
| 0.1 | 0.9992021495248345 |
| 0.3 | 0.9900787626108102 |
| 1 | 1.0380336851222152 |
| 3 | 0.9529463034831548 |
| 10 | 0.9977687831243683 |
| 30 | 0.9457203752565654 |
### Chart
| Category | |
|---|---|
| 0 | 1.0 |
| 0.1 | 0.9783775518567506 |
| 0.3 | 0.9656188355103978 |
| 1 | 0.988678186541137 |
| 3 | 0.93676869902936 |
| 10 | 0.8656019433712455 |
| 30 | 0.8258796344300355 |
### Chart
| Category | |
|---|---|
| 0 | 1.0000000000000002 |
| 0.1 | 1.0516729393327506 |
| 0.3 | 1.03390375275166 |
| 1 | 1.0134197206569124 |
| 3 | 1.0394087842371538 |
| 10 | 1.063485627016864 |
| 30 | 1.1554095297596105 |**
**
**
### Chart
| Category | |
|---|---|
| 0 | 1.0 |
| 0.1 | 0.9834825978088353 |
| 0.3 | 0.9702346280840325 |
| 1 | 1.0188427467703889 |
| 3 | 0.950714107586002 |
| 10 | 0.941987759198942 |
| 30 | 0.9709876851637919 |
### Chart
| Category | |
|---|---|
| 0 | 1.0 |
| 0.1 | 1.014927260821608 |
| 0.3 | 0.9997910851665934 |
| 1 | 1.0200734381118466 |
| 3 | 0.9549949226620965 |
| 10 | 0.9000958582684809 |
| 30 | 0.8505344451471798 |
### Chart
| Category | |
|---|---|
| 0 | 1.0 |
| 0.1 | 0.9944610564148785 |
| 0.3 | 0.9632763483755293 |
| 1 | 0.9993858133565929 |
| 3 | 1.0016691922718415 |
| 10 | 1.051718860502061 |
| 30 | 1.0249920633991967 |*
### Chart
| Category | |
|---|---|
| 0 | 1.0 |
| 0.1 | 1.0463701480249041 |
| 0.3 | 1.0810255796315997 |
| 1 | 1.164201807056732 |
| 3 | 1.1059510086549897 |
| 10 | 1.211894452876811 |
| 30 | 0.9728744262299726 |
### Chart
| Category | |
|---|---|
| 0 | 1.0 |
| 0.1 | 1.0166648684514892 |
| 0.3 | 0.9114128758876124 |
| 1 | 0.9609222199738078 |
| 3 | 0.9283955282192061 |
| 10 | 1.0277239040229087 |
| 30 | 0.9209772852279832 |
### Chart
| Category | |
|---|---|
| 0 | 1.0 |
| 0.1 | 1.0619808458834454 |
| 0.3 | 1.0022503090889907 |
| 1 | 1.116348774726933 |
| 3 | 1.0077984315177 |
| 10 | 1.0450876758525138 |
| 30 | 0.9376357595156696 |**
*

## Slide 6
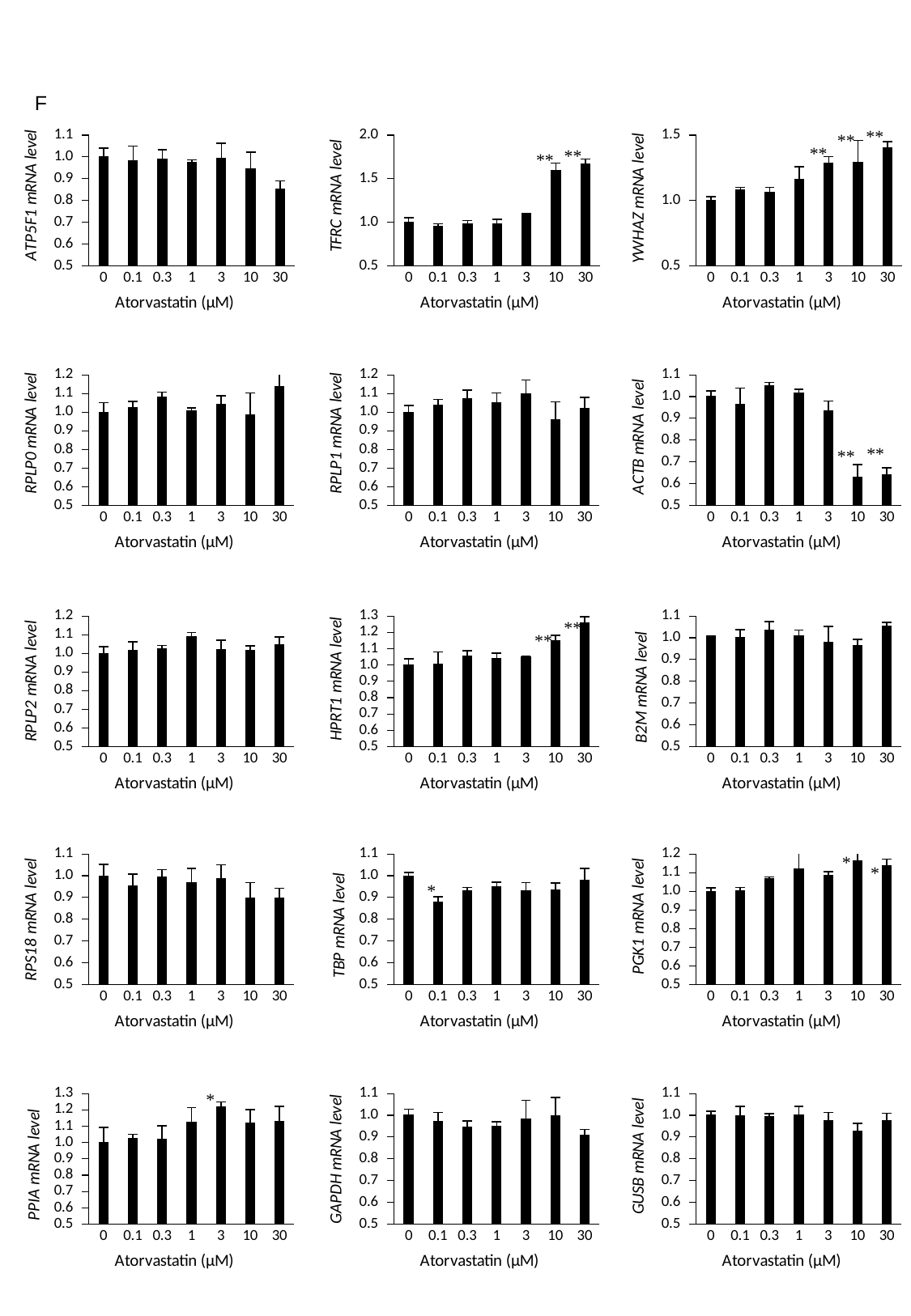

F
**
**
### Chart
| Category | |
|---|---|
| 0 | 1.0 |
| 0.1 | 0.9816426694735814 |
| 0.3 | 0.9894782028909272 |
| 1 | 0.9755130109477896 |
| 3 | 0.9949831856797271 |
| 10 | 0.9468237738773508 |
| 30 | 0.8538411733271767 |
### Chart
| Category | |
|---|---|
| 0 | 1.0 |
| 0.1 | 0.9511272792802563 |
| 0.3 | 0.9785874753944318 |
| 1 | 0.9844012401713353 |
| 3 | 1.0870888186549348 |
| 10 | 1.5993330228798956 |
| 30 | 1.6736650817575942 |
### Chart
| Category | |
|---|---|
| 0 | 1.0 |
| 0.1 | 1.0789338796788794 |
| 0.3 | 1.0660754881156065 |
| 1 | 1.1598341622462505 |
| 3 | 1.283704960266483 |
| 10 | 1.2951801192626176 |
| 30 | 1.4012692987209456 |**
**
**
### Chart
| Category | |
|---|---|
| 0 | 1.0 |
| 0.1 | 1.022582110649186 |
| 0.3 | 1.080848867411423 |
| 1 | 1.0056279076150352 |
| 3 | 1.0398176199653315 |
| 10 | 0.9857545835165662 |
| 30 | 1.137903246123743 |
### Chart
| Category | |
|---|---|
| 0 | 1.0 |
| 0.1 | 1.0376540793810165 |
| 0.3 | 1.0713410205295941 |
| 1 | 1.0513101801813063 |
| 3 | 1.0984465773392917 |
| 10 | 0.9600968482113817 |
| 30 | 1.0217611547651264 |
### Chart
| Category | |
|---|---|
| 0 | 1.0 |
| 0.1 | 0.9632207168108037 |
| 0.3 | 1.0509669550532732 |
| 1 | 1.015911013044136 |
| 3 | 0.9352916053194353 |
| 10 | 0.6320566113734395 |
| 30 | 0.6419572675303392 |**
**
### Chart
| Category | |
|---|---|
| 0 | 1.0 |
| 0.1 | 1.0158625453647037 |
| 0.3 | 1.0245766156685174 |
| 1 | 1.0899410723077883 |
| 3 | 1.0205154090759005 |
| 10 | 1.0154867584394272 |
| 30 | 1.0443027620339007 |
### Chart
| Category | |
|---|---|
| 0 | 1.0 |
| 0.1 | 1.0039011492574839 |
| 0.3 | 1.055338776460853 |
| 1 | 1.0387382715594347 |
| 3 | 1.0495372349110699 |
| 10 | 1.1475453601429215 |
| 30 | 1.2590977974490496 |
### Chart
| Category | |
|---|---|
| 0 | 1.0 |
| 0.1 | 1.0032651921820122 |
| 0.3 | 1.0347686688690125 |
| 1 | 1.009801707362685 |
| 3 | 0.979653379779715 |
| 10 | 0.9644906403722427 |
| 30 | 1.0553424413528651 |**
**
### Chart
| Category | |
|---|---|
| 0 | 1.0 |
| 0.1 | 0.953117033989464 |
| 0.3 | 0.9948480850917004 |
| 1 | 0.9682075650178718 |
| 3 | 0.9853832782772903 |
| 10 | 0.8993413263029257 |
| 30 | 0.8992386378552572 |
### Chart
| Category | |
|---|---|
| 0 | 0.9999999999999999 |
| 0.1 | 0.8790905447100927 |
| 0.3 | 0.9301642550936883 |
| 1 | 0.9489470581314432 |
| 3 | 0.9325548187138838 |
| 10 | 0.9339105188193496 |
| 30 | 0.979134397194355 |
### Chart
| Category | |
|---|---|
| 0 | 1.0 |
| 0.1 | 1.0010838686162804 |
| 0.3 | 1.065893374671638 |
| 1 | 1.1204769842525708 |
| 3 | 1.0833934729904942 |
| 10 | 1.165367206217839 |
| 30 | 1.1363387188325353 |*
*
*
### Chart
| Category | |
|---|---|
| 0 | 1.0 |
| 0.1 | 1.024951110659188 |
| 0.3 | 1.022046653172427 |
| 1 | 1.124261120475877 |
| 3 | 1.2175414900664736 |
| 10 | 1.120624041904213 |
| 30 | 1.1281140748964091 |
### Chart
| Category | |
|---|---|
| 0 | 1.0 |
| 0.1 | 0.9713969545792986 |
| 0.3 | 0.9448785811533157 |
| 1 | 0.9514376710879167 |
| 3 | 0.9835318222237891 |
| 10 | 0.9995238520153872 |
| 30 | 0.9076615986791761 |
### Chart
| Category | |
|---|---|
| 0 | 1.0 |
| 0.1 | 0.9970061769472043 |
| 0.3 | 0.9960658712713952 |
| 1 | 1.0033264914901303 |
| 3 | 0.9750179588051049 |
| 10 | 0.9273854487248904 |
| 30 | 0.9769526601213125 |*
