# Supplementary material for: Expression of housekeeping genes varies depending on mevalonate pathway inhibition in cancer cells
Source: Heliyon. 2023 Jul 8;9(7):e18017. doi: 10.1016/j.heliyon.2023.e18017 (PMC10368838; doi:10.1016/j.heliyon.2023.e18017)
Supplement: Multimedia component 3 [file mmc3.docx]

**Figure legends**

**Figure S1.** **mRNA expression levels of 15 housekeeping genes in statin-sensitive and statin-resistant cancer cells treated with atorvastatin for 24 h.**

The mRNA expression levels of 15 housekeeping genes commonly used as reference genes in statin-sensitive HOP-92 cells (**A**), statin-resistant NCI-H322M cells (**B**), statin-sensitive PC-3 cells (**C**), statin-resistant DU-145 cells (**D**), statin-sensitive SK-MEL-5 cells (**E**), and statin-sensitive MDA-MB-435 cells (**F**). Measurements of the experimental group were compared with those of the control using Dunnett’s test. Data are presented as the mean ± standard deviation of triplicate samples. **P* < 0.05, ***P* < 0.01, with respect to each control group.
